# Genetic diversity and zoonotic transmission potential of *Blastocystis* sp. in Southeast Asia: A scoping review of molecular evidence

**DOI:** 10.1016/j.parepi.2026.e00492

**Published:** 2026-03-12

**Authors:** Picha Suwannahitatorn

**Affiliations:** Department of Parasitology, Phramongkutklao College of Medicine, Bangkok 10400, Thailand

**Keywords:** *Blastocystis* sp., Zoonotic potential, Epidemiology, Subtypes, Irritable bowel syndrome, Southeast Asia

## Abstract

*Blastocystis* sp. is a biological paradox, functioning as both a prevalent gut inhabitant and a potential pathogen. This scoping review synthesizes molecular evidence on the genetic diversity and zoonotic transmission dynamics of this species across Southeast Asia. Following PRISMA-ScR guidelines, we analyzed 61 molecular studies published through November 15, 2025, to map the organism's regional footprint. The results characterize *Blastocystis* sp. as a genetically diverse species complex deeply embedded in the region's human-animal interface. While ST1 and ST3 are the predominant human subtypes, Southeast Asia exhibits distinct epidemiological patterns driven by zoonotic pressure. Avian subtypes (ST6 and ST7) show unusually high prevalence in the Philippines and Indonesia, suggesting active spillover from poultry. Similarly, ST1 infections in Thailand are genetically linked to porcine reservoirs, highlighting the role of livestock and organic fertilizers in transmission. Water sources also act as critical environmental sinks for diverse subtypes (ST1–ST4), facilitating community-wide exposure. Regarding clinical significance, pathogenicity appears context-dependent rather than intrinsic. Statistical analyses associate ST1 and ST3 with Irritable Bowel Syndrome (IBS) and chronic urticaria. Conversely, other subtypes, such as ST4, are often associated with healthy gut microbiomes. Current evidence supports a passenger hypothesis, suggesting that routine treatment might not be justified based on current molecular data. However, the substantial overlap of subtypes between humans, livestock, and companion animals underscores the need for One Health-oriented control strategies. Future research must prioritize longitudinal studies to clarify when this common colonizer shifts from a commensal passenger to a driver of disease.

## 1 Background

### 1.1 The taxonomic and biological paradox

Among the countless organisms living in the human gastrointestinal tract, *Blastocystis* sp. stands out as a biological paradox. It is both one of the most common eukaryotic inhabitants of the human gut and one of the least understood (Aykur et al., 2024). For more than a century, clinicians and researchers have struggled to categorize this organism definitively. The dichotomy, whether it is a pathogen that needs to be eradicated or a harmless commensal indicating a healthy microbiome, reflects the current state of *Blastocystis* sp. research (Aykur et al., 2024).

The history of *Blastocystis* sp. has been marked by mistaken identity. When Brumpt first characterized the organism in 1912, the scientific community lacked the molecular tools needed for accurate classification. Over the years, it was incorrectly categorized as a flagellate cyst, a yeast, a fungus, or even a plant fragment due to its variable appearances (Zierdt, 1991). It was only through extensive research by Zierdt et al. that the protozoan nature of the organism was finally confirmed. Zierdt showed that *Blastocystis* sp. did not grow on fungal media and was sensitive to antiprotozoal drugs, clearly distinguishing it from yeasts and fungi (Zierdt, 1991).

However, modern molecular phylogenetics has further advanced our understanding. We now know that *Blastocystis* sp. is not a typical protozoan but belongs to the Stramenopiles (heterokonts) (Stensvold et al., 2007). This classification places it in a distinct evolutionary lineage related to brown algae, diatoms, and water molds rather than flagellates or amoebae, which are usually found in the gut (Stensvold et al., 2007). This unique taxonomic position may explain why *Blastocystis* sp. exhibits such unusual biological behaviors, including the presence of mitochondrion-like organelles, and variable responses to standard antimicrobial treatments (Stensvold, 2013).

### 1.2 The genetic revolution: from morphology to subtypes

The most crucial factor in understanding the clinical behavior of *Blastocystis* sp. is its significant genetic diversity. The genus is not uniform; it includes a vast array of distinct genetic isolates called subtypes (STs). Early research identified a handful of subtypes (Nemati et al., 2021), but recent molecular analyses using Small Subunit ribosomal RNA (SSU rRNA) gene sequencing have revealed at least 44 distinct subtypes (Cakir et al., 2019). The genetic differences between these subtypes are substantial, often exceeding the genetic distance between distinct species in other genera, suggesting they likely represent distinct species with unique biological traits and host interactions (Stensvold, 2013; Riswana et al., 2025).

Of these 44+ subtypes, approximately 17 (ST1–ST10, ST12, ST14, ST16, ST23, ST26, ST35, ST41) have been linked to human infections. However, their distribution is not random. Globally, ST1, ST2, ST3, and ST4 account for over 90% of human carriage (Stensvold, 2013). Different subtypes appear to have distinct reservoirs and clinical implications. For example, ST3 is the most common human subtype worldwide, whereas ST4 is highly prevalent in Europe but historically rare in Southeast Asia (Stensvold et al., 2007). This genetic diversity suggests that when we refer to “*Blastocystis* sp.,” we are essentially referring to a species complex with varying potential to cause disease, act as a passenger, or coexist peacefully with hosts.

### 1.3 The “passenger” hypothesis and clinical uncertainty

The debate over the pathogenicity of *Blastocystis* sp. has evolved beyond the binary “pathogen vs. commensal” argument. Heneberg (2025) proposed the “Passenger Hypothesis,” suggesting that *Blastocystis* sp. is a context-dependent component of the gut ecosystem (Heneberg, 2025a). In this view, the organism is neither inherently good nor bad but reflects the host's ecological state. Its presence often correlates with high bacterial diversity and a healthy microbiome (Nieves-Ramírez et al., 2018), yet in specific contexts defined by host immune status, microbiome dysbiosis, or subtype virulence (e.g., ST1, ST3), it may contribute to disease (Riswana et al., 2025). This scoping review adopts this nuanced framework to analyze the molecular evidence from Southeast Asia.

## 2 Methods

This focused scoping review was designed to map the existing literature on *Blastocystis* sp., specifically examining the intersection of genetic diversity, clinical manifestations, and regional epidemiology in Southeast Asia. The review protocol follows the PRISMA-ScR (Preferred Reporting Items for Systematic Reviews and Meta-Analyses extension for Scoping Reviews) guidelines to ensure that the evidence synthesis remains thorough, transparent, and reproducible.

### 2.1 Search strategy and selection process

A systematic search strategy was developed to identify all relevant peer-reviewed literature published through November 15, 2025. Four major electronic databases were searched: PubMed, Scopus, Web of Science, and Google Scholar. Web of Science was included to ensure comprehensive coverage of high-impact systematic reviews, while Google Scholar served as a supplementary source to capture gray literature and regional Southeast Asian journals not indexed in the primary databases. The search algorithms combined Medical Subject Headings (MeSH) and free-text terms to capture the organism's diverse features. The core search strings and the number of articles found in each database are shown in Table 1.

**Table 1:** Specific algorithms and keywords for the identification of relevant literature in databases.

Database	Specific Keywords / Search Algorithms	Records Found
PubMed	(“*Blastocystis*” OR “*Blastocystis hominis*” OR “*Blastocystis* sp.”) AND (“subtype” OR “genotype” OR “PCR” OR “sequencing” OR “molecular”) AND (“Southeast Asia” OR “Thailand” OR “Malaysia” OR “Vietnam” OR “Philippines” OR “Indonesia” OR “Singapore” OR “Cambodia” OR “Myanmar” OR “Laos” OR “Timor-Leste”)	152
Web of Science		145
Scopus	TITLE-ABS-KEY ((“*Blastocystis*” OR “*Blastocystis* sp.”) AND (“genetic diversity” OR “zoonosis” OR “epidemiology”) AND (“Southeast Asia” OR [List of Countries]))	188
Google Scholar	*Blastocystis* subtypes Southeast Asia molecular epidemiology	80
**Total**		565

**Fig. 1:**
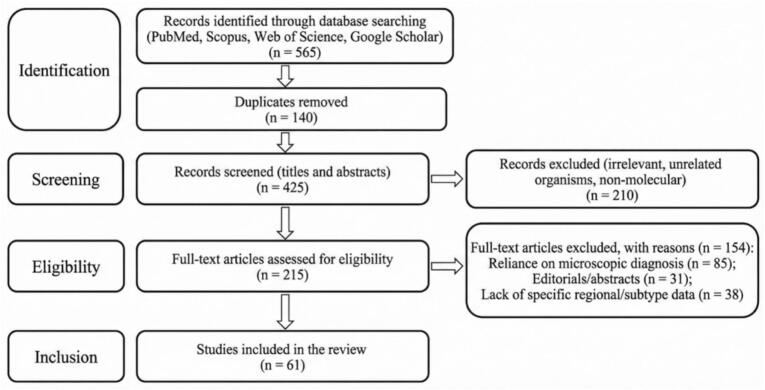
PRISMA flowchart.

As shown in Fig. 1, the systematic literature search across databases initially yielded 565 records. After removing 140 duplicates, 425 unique citations underwent title and abstract screening, resulting in the exclusion of 210 records deemed irrelevant or non-molecular. Of the 215 full-text articles subsequently assessed for eligibility, 154 were excluded based on rigorous criteria: 85 relied exclusively on microscopic diagnosis, 31 were limited to editorials or abstracts, and 38 lacked specific regional data or subtype differentiation. Consequently, 61 peer-reviewed molecular studies met all inclusion requirements and were selected for this review.

### 2.2 Inclusion and exclusion criteria

We applied strict inclusion criteria to select studies that provided meaningful primary data or high-level syntheses relevant to the research questions. Original research articles using molecular methods (PCR, sequencing) to detect and subtype *Blastocystis* sp.; systematic reviews and meta-analyses; studies exploring zoonotic transmission pathways (animal reservoirs); and clinical studies linking infection to symptoms (IBS, urticaria) with molecular confirmation. Studies relying solely on microscopic diagnosis (smear/culture) were excluded because microscopy cannot differentiate subtypes, which is the central focus of this review. We acknowledge that this strict criterion excludes some rural epidemiological data, potentially lowering overall prevalence estimates, but it ensures the high specificity needed to analyze subtype-dependent pathogenicity. Studies focused on non-mammalian hosts without zoonotic implications were also excluded.

Because of differences in how metadata is indexed across databases, duplicate detection was conducted using strict algorithmic matching criteria. A complete list of the initial 565 raw search results is included in Supplementary Table S1 to ensure transparency in the deduplication process.

### 2.3 Quality assessment

Although quality assessment is generally optional in scoping reviews, we evaluated the diagnostic accuracy of the included studies. We prioritized studies that used DNA sequencing of the SSU rRNA gene, as this is the gold standard for subtyping. Studies using only sequence-tagged site (STS) primers were included but interpreted with caution because of potential cross-reactivity or limited subtype coverage. This assessment ensured that conclusions regarding subtype distribution (e.g., ST1 vs ST3 dominance) were based on data with high internal validity.

### 2.4 Evidence extraction and analysis

Data extraction was performed to synthesize findings from the diverse studies included. We systematically gathered key variables: geographic location, sample size, diagnostic methods, prevalence ranges, dominant subtypes (ST1–ST44), animal reservoirs, and statistical associations with clinical symptoms. The analysis involved a narrative synthesis, organizing findings into thematic categories: taxonomy, epidemiology, pathogenicity, and zoonotic potential.

## 3 Results

### 3.1 Microbiology and genetic diversity

#### 3.1.1 Morphological heterogeneity

The wide morphological variability of *Blastocystis* sp. has long made accurate classification difficult. Instead of a single shape, the organism transitions through four distinct forms: vacuolar, granular, amoeboid, and cyst (Roberts et al., 2011). The most commonly observed stage in clinical stool samples is the vacuolar form, characterized by a large central vacuole (8–10 μm) (Wawrzyniak et al., 2013). The amoeboid form is often cited as the invasive stage, potentially interacting with the host immune system. The cyst form (3–5 μm) is the transmissible stage, is resistant to environmental stress and standard chlorination, and facilitates waterborne transmission (Zierdt, 1991).

#### 3.1.2 The subtype ecosystem

*Blastocystis* sp. comprises at least 44 subtypes. In Southeast Asia, the subtype landscape is dominated by ST1, ST3, and ST2, but the diversity is richer than in Western regions. Table 2 summarizes the characteristics of the medically relevant subtypes found in the region.

**Table 2:** Medically Relevant *Blastocystis* sp. Subtypes (STs) in Southeast Asia.

Subtype (ST)	Regional Prevalence (SEA)	Primary Animal Reservoir(s)	Reported Association with Human Disease
ST1	High	Pigs, Cattle, Dogs, Primates	Strongly associated with IBS (OR 4.40) and zoonotic transmission from livestock (Riswana et al., 2025; Rostami et al., 2017)
ST2	Moderate	Primates, Pigs, Dogs	Linked to urticaria; high genetic variability suggests polyphyletic origin (Riswana et al., 2025)
ST3	High / Dominant	Humans, Primates, Dogs, Cattle	Most common human ST; often associated with human-to-human transmission (Rostami et al., 2017)
ST4	Low / Emerging	Rodents, Cats	Traditionally rare in Asia; associated with healthy gut flora but also found in rodents (Stensvold et al., 2007; Nieves-Ramírez et al., 2018)
ST5	Low	Pigs	Strongly zoonotic; rare in humans, serves as a marker for pig exposure (Riswana et al., 2025)
ST6	Moderate (Regionally)	Birds (Poultry)	High prevalence in Philippines/Indonesia indicates active poultry-to-human transmission (Riswana et al., 2025; Belleza et al., 2016)
ST7	Moderate (Regionally)	Birds (Poultry)	Avian subtype found in humans; linked to gastrointestinal symptoms (Riswana et al., 2025; Rostami et al., 2017)
ST10	Low	Cattle, Sheep	Emerging zoonotic subtype found in livestock and occasional human cases (Rostami et al., 2017)

### 3.2 Epidemiology and transmission dynamics

#### 3.2.1 Regional epidemiology: the southeast Asian context

Southeast Asia (SEA) has some of the highest *Blastocystis* sp. prevalence globally. However, molecular data reveal significant heterogeneity. Unlike microscopy-based surveys, which often overestimate prevalence due to artifacts, molecular surveys provide a clearer, albeit sometimes lower, estimate of true infection rates (Riswana et al., 2025). As shown in Table 3, we report prevalence ranges rather than averages to account for study heterogeneity (e.g., hospital vs. community).

**Table 3:** Molecular Epidemiology of *Blastocystis* sp. in Southeast Asian Countries.

Country	Molecular Prevalence (%)	Dominant Human Subtypes	Key Animal Reservoirs & Notes	References
Philippines	15.3%–82.9%	ST3, ST1, ST6	Highest regional prevalence; high detection of ST6 and ST7 (avian subtypes) suggests intense zoonotic pressure from poultry farming.	(Riswana et al., 2025; Belleza et al., 2016; Adao et al., 2016; Rivera, 2008)
Indonesia	29.9%–33.8%	ST1, ST3, ST7	ST7 presence (8.5%) links to bird sources; high burden in rural communities with poor sanitation.	(Yoshikawa et al., 2016; Kesuma et al., 2019)
Thailand	5.2%–40.6%	ST3, ST1, ST4	Pigs are major reservoirs (76.4% infection rate); high rates reported in military personnel and orphans.	(Kotepui et al., 2025; Jantermtor et al., 2013; Thathaisong et al., 2013; Pintong et al., 2018; Udonsom et al., 2018; Pipatsatitpong et al., 2015; Popruk et al., 2015; Wongthamarin et al., 2020; Srichaipon et al., 2019)
Malaysia	9.2%–40.3%	ST3, ST1, ST2	Extensive diversity found in water catchments (ST1–ST4); broad host range identified in peri-domestic animals.	(Noradilah et al., 2016; Tan et al., 2009; Tan et al., 2013)
Singapore	3.30%	ST3	Low prevalence reflects high urbanization and sanitation; dominance of ST3 implies a human-to-human transmission cycle.	(Wong et al., 2008)
Vietnam	Limited Data	ST1, ST3	Emerging molecular data suggests potential waterborne transmission links in schoolchildren.	(Nguyen et al., 2023)

**Thailand:**

A systematic review and meta-analysis of Thai community studies (2025) noted significant disparities in prevalence estimates across the country (Kotepui et al., 2025). Military personnel (29.9%) and orphans (29.0%) have much higher rates, likely due to crowded living conditions and shared water sources (Thathaisong et al., 2013; Pipatsatitpong et al., 2015). Subtype analysis confirms ST3 and ST1 as the primary lineages, with ST1 being significantly associated with pig farming in rural provinces (Jantermtor et al., 2013; Wongthamarin et al., 2020; Ruang-Areerate et al., 2021).

**Philippines and Indonesia:**

These nations illustrate the “avian connection.” The unusually high prevalence of ST6 and ST7 (together ∼15% in some Philippine cohorts) contrasts sharply with global trends, where these subtypes are rare (<1%) (Rivera, 2008). This suggests a specific transmission dynamic involving close contact with poultry, a common practice in rural households in these archipelagos (Rivera, 2008).

**Malaysia and others:**

Studies in Malaysia have documented prevalence in rural communities (Mohammad et al., 2017), among cancer and HIV patients (Tan et al., 2009), and in livestock, including goats (Tan et al., 2013). Similarly, cross-border studies involving Cambodia have identified ST1 and ST5 in pigs and in-contact humans (Wang et al., 2014).

#### 3.2.2 Zoonotic transmission potential

The “One Health” perspective is critical for *Blastocystis* sp. The overlap of subtypes between humans and animals indicates a high potential for zoonotic transmission, though distinguishing active transmission from shared environmental exposure remains challenging.

**Livestock (Pigs and Cattle):**

Livestock are major reservoirs. In Thailand, pigs carrying ST1, ST3, and ST5 serve as sources of environmental contamination, and human ST1 infections genetically identical to those in local pigs have been linked to the use of pig feces as organic fertilizer (Jantermtor et al., 2013; Thathaisong et al., 2013). A recent study in Iran (relevant to the global context) confirmed that cattle are primary hosts for ST10 and ST14, and that sheep are hosts for ST10, ST3, and ST2 (Shams et al., 2024). The identification of ST10 and ST14 in humans suggests these are neglected zoonotic subtypes (Rostami et al., 2017; Mohammad et al., 2017). Dairy calves have also been identified as reservoirs for diverse subtypes (Maloney et al., 2019).

**Companion animals (Dogs and Cats):**

Often overlooked, pets are significant reservoirs. Studies have shown that domestic animals in contact with humans serve as critical nodes in transmission cycles (Mahdavi et al., 2022). Shams et al. (2024) provide the first molecular characterization of subtypes in domestic animals in Western Iran, identifying genetic overlap between human and animal isolates and further highlighting the zoonotic risk in domestic environments (Mahdavi et al., 2022). Crucially, dogs predominantly carry ST1, ST2, and ST3, the same subtypes dominant in humans. Cats are more likely to carry ST4 and ST10. Phylogenetic analysis shows high genetic similarity between human and canine isolates of ST3, supporting the potential for bidirectional transmission in households (Heneberg, 2025b). Geographic surveys have also detected ST1-ST4 in dogs (Wang et al., 2013).

**Non-human primates:**

Studies in macaques support the role of non-human primates as reservoirs for zoonotic subtypes (Li et al., 2020).

**Avian reservoirs:**

Poultry are the natural hosts for ST6 and ST7. The recovery of these subtypes from human stool in SEA is a strong indicator of zoonotic transmission (Belleza et al., 2016; Rivera, 2008).

**Waterborne transmission:**

Water is a key abiotic reservoir (Attah et al., 2023; Lee et al., 2012). A 2024 systematic review by Mahdavi et al. found *Blastocystis* sp. in 35.5% of wastewater and 19.1% of tap water samples worldwide (Mahdavi et al., 2024). Nine subtypes (ST1–ST4, ST6, ST8, ST10, ST21, ST24) have been identified in water, confirming water as a vehicle for diverse zoonotic subtypes. In Malaysia, river water analysis identified ST1, ST2, ST3, and ST4, mirroring the human distribution (Noradilah et al., 2016). In Thailand, infection rates in schoolchildren have been linked to drinking water quality (Leelayoova et al., 2008).

### 3.3 Pathogenicity versus commensalism

The clinical significance of *Blastocystis* sp. remains one of the most contentious issues in parasitology. Current evidence supports a “Context-Dependent” model rather than a strict pathogen definition.

#### 3.3.1 The case for pathogenicity

Evidence suggests that *Blastocystis* sp. can act as a pathogen under specific conditions. Virulence studies have identified cysteine proteases secreted by the organism that can hydrolyze secretory IgA (sIgA) and degrade zonulin, a protein that regulates gut permeability (Rojas-Velázquez et al., 2022). This disruption (“leaky gut”) can trigger inflammation. Statistical data support this: ST1 and ST3 are significantly more common in symptomatic patients. A meta-analysis found that ST1 had an Odds Ratio (OR) of 4.40 for association with Irritable Bowel Syndrome (IBS) (Rostami et al., 2017; Cifre et al., 2018). Similarly, ST3 has been linked to chronic urticaria, with case reports symptom improvement associated with the clearance of the parasite (Bahrami et al., 2020). Other studies confirm significantly higher carriage in IBS patients compared to controls (Salvador et al., 2021; Kumarasamy et al., 2023).

#### 3.3.2 The case for commensalism and the “passenger hypothesis”

Conversely, high prevalence in healthy individuals supports the commensal view. Heneberg (2025) argues that *Blastocystis* sp. is often a “passenger” in a healthy ecosystem (Heneberg, 2025a). Colonization is frequently linked to increased bacterial alpha-diversity and the presence of beneficial bacteria such as *Faecalibacterium prausnitzii* (Nieves-Ramírez et al., 2018; Olyaiee et al., 2022). In this framework, the organism requires a healthy gut to thrive. The association with disease in some patients might be due to specific virulent strains or a “second hit” (e.g., co-infection, stress) that shifts the organism from passenger to pathogen (Deng et al., 2021; Noradilah et al., 2017). Emerging research suggests that *Blastocystis* sp. may even influence the gut-brain axis through tryptophan pathways (Deng and Tan, 2025).

#### 3.3.3 Subtype-specific effects

The solution to the paradox likely lies in subtype specificity. For instance, ST4 is often linked to healthy gut flora in Europeans and shows low genetic diversity, suggesting recent expansion (Rudzińska and Sikorska, 2023). In contrast, ST1 and ST3 show high genetic variability and are more frequently implicated in dysbiosis and inflammation in Asian populations.

### 3.4 Clinical manifestations

Symptomatic *Blastocystosis* presents a non-specific profile.

**Irritable Bowel Syndrome (IBS):**

The strongest link is with the IBS-Diarrhea type. The mechanism likely involves low-grade inflammation driven by protease secretion (Cifre et al., 2018).

**Dermatological conditions:**

Chronic urticaria (hives) is the most common extra-intestinal manifestation (Bahrami et al., 2020). The proposed mechanism is cutaneous immune activation secondary to disruption of the gut barrier.

### 3.5 Diagnostic strategies

Microscopy yields low sensitivity (∼48%) and cannot distinguish subtypes (Roberts et al., 2011). Culture increases sensitivity but is labor-intensive (Jones' medium) (Boutahar et al., 2023). PCR/sequencing is the gold standard. Essential for subtyping and determining zoonotic potential (Wakid et al., 2022). Barcoding of the SSU rRNA gene is the preferred method (Centers for Disease Control and Prevention, 2019).

### 3.6 Implications for clinical research

Conflicting evidence regarding pathogenicity requires a conservative clinical approach. Routine treatment is not recommended for asymptomatic carriers (Sekar and Shanthi, 2013). Eradication may disrupt the gut microbiome and offers no proven benefit (Stensvold et al., 2009). For symptomatic patients, treatment should be considered only after other causes are ruled out (Centers for Disease Control and Prevention, 2024). Metronidazole has been the historical first-line drug, but reports of variable efficacy and potential resistance are increasing (Sekar and Shanthi, 2013; Roberts et al., 2015). Nitazoxanide and Trimethoprim-Sulfamethoxazole (TMP-SMX) are alternatives (Sekar and Shanthi, 2013). *Saccharomyces boulardii* (probiotic) shows promise in reducing symptoms, possibly by restoring microbiome balance rather than eradicating the parasite (Aykur et al., 2024).

## 4 Discussion and conclusion

### 4.1 Interpretive framework

Molecular data from Southeast Asia reinforce the view of *Blastocystis* sp. as a complex biological entity. The “ecosystem modulator” or “passenger” model offers a better fit for the epidemiological data than the strict pathogen model. The high prevalence in healthy populations in the Philippines and Thailand suggests that colonization is benign for many. However, the statistical link between ST1/ST3 and IBS cannot be ignored, suggesting that pathogenicity is an emergent property of specific host-subtype interactions.

### 4.2 Zoonotic implications

The identification of “avian” (ST6, ST7) and “livestock” (ST1, ST3, ST5, ST10) subtypes in humans confirms that *Blastocystis* sp. transmission in Southeast Asia is deeply embedded in the human-animal interface. Recent findings of ST1 and ST3 in pet dogs (Heneberg, 2025b) and livestock underscore the need for One Health-oriented control strategies. Water and sanitation are paramount, as water sources serve as sinks for diverse subtypes of human and animal waste.

### 4.3 Limitations and gap analysis

Several limitations should be considered when interpreting the findings of this scoping review. Methodologically, the literature search, study selection, and data extraction processes were carried out by a single author. Although strict adherence to predefined inclusion and exclusion criteria was maintained, the lack of a second independent reviewer introduces a potential risk of selection bias and reduces the ability to systematically validate the extracted data, which is a common limitation in scoping review methodologies.

Significant gaps remain. There is a lack of molecular data for Laos, Cambodia, and Myanmar. Most studies are cross-sectional, which prevents causal inference. Longitudinal studies are needed to track subtype stability over time and determine whether “passenger” colonization can progress to pathogenicity.

### 4.4 Conclusion

*Blastocystis* sp. in Southeast Asia is a genetically diverse species complex with significant zoonotic transmission potential. The organism ranges from a commensal passenger in healthy individuals to a potential driver of gastrointestinal symptoms in specific contexts. Molecular surveillance indicates that transmission is driven by overlap with pigs, poultry, and companion animals, as well as by waterborne routes. Clinical management should be cautious, avoiding routine treatment of asymptomatic carriers, while public health efforts should focus on sanitation and reservoir control to mitigate the burden posed by potentially pathogenic subtypes (ST1, ST3).

## Personal and professional relationships

The author confirms there are no personal or professional relationships with individuals or organizations whose interests could be positively or negatively impacted by the publication of the manuscript.

## CRediT authorship contribution statement

**Picha Suwannahitatorn:** Writing – review & editing, Writing – original draft, Validation, Methodology, Formal analysis, Data curation, Conceptualization.

## Funding

This research did not receive any specific funding from public, commercial, or non-profit organizations. The work was conducted solely with the resources available to the author.

## Declaration of competing interest

The following is a detailed disclosure of any potential financial, professional, or personal relationships that might be seen as influencing the work reported in the above-titled manuscript.

**Financial Interests:**

• The author confirms that they have not received any payments or services from third parties, such as commercial entities, non-profit organizations, or government agencies, that could be considered a financial interest in the results or conclusions of this manuscript.
• The author has no stocks or shares in any organization that could financially benefit or be harmed by the publication of this research.
• There are no patent applications or royalties associated with this work.

## Appendix A Supplementary data

Supplementary material Image 1
