# Supplementary material for: Genetic diversity and zoonotic transmission potential of Blastocystis sp. in Southeast Asia: A scoping review of molecular evidence
Source: Parasite Epidemiol Control. 2026 Mar 12;33:e00492. doi: 10.1016/j.parepi.2026.e00492 (PMC13010985; doi:10.1016/j.parepi.2026.e00492)
Supplement: Supplementary file 1 — Supplementary material [file mmc1.pdf]

S/N	Title	Authors	Year	Journal	Search Source
1	Prevalence and subtype distribution of Blastocystis infections among community participants in Thailand: a systematic review and meta-analysis	Kotepui M, Popruk S, Kotepui KU, Masangkay FR, Wangdi K, Mahittikorn A, Stensvold CR	2025	Parasite	Web of Science
2	Prevalence of Blastocystis infection in patients with colorectal cancer in Taiwan.	Liao, Chen, Tsai, Cheang, Lin, Chen, Chen, Chu	2025	BMC infectious diseases	Google Scholar
3	Prevalence of Intestinal Protozoa Among Patients Living with HIV in the Peruvian Amazon.	Otero-Rodriguez, Pinedo-Cancino, Casapia-Morales, Villacorta-Pezo, Mego-Campos, Parraguez-de-la-Cruz, Merino, Clark, Ramos-Rincon	2025	Tropical medicine and infectious disease	PubMed
4	First molecular identification and genetic diversity of Giardia duodenalis and Blastocystis sp. in patients with multiple sclerosis in Iran.	Shafiee, Modiri, Khademerfan, Bahrami	2025	Molecular biology reports	Scopus
5	Identifying actionable genetic mutations and microsatellite instability in liquid biopsy of colorectal cancer.	Kassem, Seadawy, Gaafar, Kassem, Hussein A, Rizk A, Hassan, AbdelAziz, Abdelradi, El-Hosseny, Abdulla, Moneim	2025	Journal, genetic engineering & biotechnology	Scopus
6	A protocol for mapping Blastocystis epidemiology and diagnostics from One Health perspective	Akdur Ozturk, E., Guadano-Procesi, I., Figueiredo, A. M., Godfrey, A., Gentekaki, E., Tsaousis, A. D., Carmena, D., & Dogruman-Al, F	2025	Open research Europe	Web of Science
7	Genetic characterization of enteric protozoan microorganisms in newly arrived migrants in Italy and correlation with the gut microbiome layout.	Marangi, Palladino, Valzano, Scicchitano, Turroni, Rampelli, Candela, Arena	2025	Travel medicine and infectious disease	Scopus
8	The Role of Blastocystis spp. in the Etiology of Gastrointestinal and Autoimmune Diseases	Pawelec-Pyciak O, Lanocha-Arendarczyk N, Grzeszczak K, Kosik-Bogacka D	2025	Pathogens	Web of Science

S/N	Title	Authors	Year	Journal	Search Source
9	Frequency of Intestinal Protozoa in Patients Receiving Treatment at Van Special Physioactive Special Education and Rehabilitation Center.	Saygin, Aydemir, Ekici, Yilmaz	2025	Turkiye parazitolojii dergisi	Web of Science
10	The interplay between Blastocystis and human gut microbiota.	Lepczynska	2025	Annals of parasitology	Scopus
11	Infectious disease burden and surveillance challenges in Jordan and Palestine: a systematic review and meta-analysis.	Badran, Rayyan, Jaberi A, Azzam, Ramadan, Khader, Alqutob, Bakri, Qasrawi, Yacoub, Sharaqa, Fraihat, Trigui, Sokhn, Tayyem, Musa, Kong	2025	Frontiers in digital health	Scopus
12	A Systematic Review about the Efficacy of Antiparasitic Agents in the Treatment of Blastocystis Species.	Bagci U, Akarsu A	2025	Acta parasitologica	Google Scholar
13	Assessing healthcare workers as potential stool donors for faecal microbiota transplantation: a cross-sectional study of antimicrobial-resistant gut bacteria and enteropathogenic micro-organisms.	Bonilla-Moreno, M., Medina-Gomez, C., Guevara-Nunez, D., Saiz-Escobedo, L., Marti, S., Dominguez, M. A., Carrera-Salinas, A., & Rodriguez-Sevilla, G	2025	The Journal of hospital infection	PubMed
14	From parasite to partner: unravelling the multifaceted role of Blastocystis in human health and disease	Deng L, Tan KSW	2025	The Lancet microbe	Scopus
15	The impact of socioeconomic status on the burden of foodborne illnesses: a scoping review in the Middle East and North African region.	Khatib A, Kabir	2025	Frontiers in microbiology	Scopus
16	Waterborne Transmission Driving the Prevalence of Blastocystis sp. in Los Rios Region, Southern Chile	Teneo DS, Chesnais CB, Manzano J, Moll MP, Tellez A, Valenzuela-Nieto G	2025	Microorganisms	Google Scholar
17	Investigation of Blastocystis spp. in patients with inflammatory bowel disease by direct microscopy and molecular methods	Feride, Rugiyya, Huseyin, Salih	2025	African health sciences	Google Scholar

S/N	Title	Authors	Year	Journal	Search Source
18	Blastocystis infection in Tibetan antelopes (Pantholops hodgsonii) alters gut microbiota composition and function	Liu, Qin, Lei, Ma, Xie, Liu, Li, Ni, Yu, Liang, Shi, Qin, Jiang, Yan, Chen, Li, Sun	2025	Frontiers in cellular and infection microbiology	Scopus
19	Global prevalence and associated factors of intestinal parasitic infections among institutionalized populations: a systematic review and meta-analysis.	Abaka-Yawson, Squire, Issah, Ablordey, Ativi, Bawua, Arko-Mensah	2025	BMC infectious diseases	PubMed
20	Crossing the species barrier: Bidirectional transmission of Blastocystis between pets and their owners	Heneberg P	2025	One Health	Scopus
21	Detection of Blastocystis spp. in patients with urticaria and identification of subtypes using sequencing techniques	Musayeva, L., Kurtipek, G. S., Ozden, O., & Macin, S	2025	Revista Cientifica-facultad De Ciencias Veterinarias	PubMed
22	Parasite-mediated alteration of behaviour and biomolecular dynamics in a mouse model.	Leonardi, Png, Bo, Wong, Iyer R, Tan	2025	Frontiers in cellular and infection microbiology	Web of Science
23	Blastocystis and Cryptosporidium in association with biofilms in a contaminated watercourse	Estrada V, Leone M, Saura A, Farber M, Lopez-Arias L	2025	Parasitology	PubMed
24	From parasite to partner: unravelling the multifaceted role of Blastocystis in human health and disease	Deng L, Tan KSW	2025	The Lancet microbe	Google Scholar
25	Subtype Identification of Blastocystis sp. Among Humans and Domestic Animals in the West of Iran	Mohammadi P, Bozorgomid A, Mirjalali H, Javadi A, Saraei M, Heydarian P, Harandi MF, Varkiani ME, Hajialilo E	2025	Veterinary medicine and science	PubMed

S/N	Title	Authors	Year	Journal	Search Source
26	Is eosinophilia a reliable diagnostic clue for chronic strongyloidiasis? a case series from Khuzestan Province, Iran.	Ashiri, Rafiei, Ansari, Beiromvand	2025	BMC infectious diseases	Google Scholar
27	Prevalence of Blastocystis hominis, Cryptosporidium, and Strongyloidesstercoralis among patients with colorectal carcinoma in Egypt.	Gaber, Taha M, Roby, William, Mostafa, Marzaban	2025	Arab journal of gastroenterology : the official publication of the Pan-Arab Association of Gastroenterology	Web of Science
28	Molecular characterization and zoonotic potential of Blastocystis subtypes in domestic pigs and cattle from Hainan, a tropical island province in China.	Zhang, Li, Lai, Wang, Li, Ren, Yu, Li, Liu, Qiang, Li, Zhou, Lei, Wu, Zhao, Lu	2025	Parasite (Paris, France)	Web of Science
29	A Systematic Review about the Efficacy of Antiparasitic Agents in the Treatment of Blastocystis Species.	Bagci U, Akarsu A	2025	Acta parasitologica	Scopus
30	Blastocystis and Cryptosporidium in association with biofilms in a contaminated watercourse	Estrada V, Leone M, Saura A, Farber M, Lopez-Arias L	2025	Parasitology	Google Scholar
31	Intestinal Parasitic Infections, Eosinophilia, and Th1/Th2 Immune Profiles in Haemodialysis Patients.	Shafaei, Talebi, Zarean, Mosavat, Khajedaluee, Afzalaghaee, Shamsian, Ghezeldasht A	2025	Parasite immunology	Web of Science
32	Blastocystis colonization among inland and forest periphery Negritos in Malaysia: The crucial role of sanitation and hygiene practices.	Rozani, Aazmi, Attah, Termizi M, Shahrizal, Idorus, Muslim	2025	Parasitology international	PubMed
33	Synovial Parasitosis and Inflammatory Biomarker Profiles in Osteoarthritis: Associations with Host and Therapeutic Factors.	Hassanein, Shehata, Aldawoudy, Masoud	2025	Acta parasitologica	Scopus

S/N	Title	Authors	Year	Journal	Search Source
34	Anti-inflammatory effect of probiotics in patients with Blastocystis spp. infection.	Abdeltawab, Aal A, Ali, Zaki, El-Din S, Amin	2025	Cytokine	Google Scholar
35	Aspects of Genetic Diversity, Host Specificity and Public Health Significance of Single-Celled Intestinal Parasites Commonly Observed in Humans and Mostly Referred to as 'Non-Pathogenic'.	Stensvold	2025	APMIS : acta pathologica, microbiologica, et immunologica Scandinavica	Scopus
36	Molecular detection of zoonotical and non-zoonotical Blastocystis subtypes in domestic and wild animals from the Valencian Community (Spain)	Marti-Marco, Moratal, Torres-Blas, Cardells, Lizana, Dea-Ayuela	2025	Frontiers in veterinary science	Scopus
37	Assessment of the Therapeutic Role of Allium tuncelianum Extract in Rats Infected with Blastocystis Subtype 3.	Aykur, Tosun G, ozgur	2025	Acta parasitologica	Web of Science
38	Investigation of Antiparasitic Effect of Juniperus communis L. Fruits Extracts	cavus, ozel, Tunali, Kayalar, Yereli, ozbilgin	2025	Turkiye parazitolojii dergisi	Google Scholar
39	Detection of Blastocystis spp. in patients with urticaria and identification of subtypes using sequencing techniques	Musayeva, L., Kurtipek, G. S., Ozden, O., & Macin, S	2025	Revista Cientifica-facultad De Ciencias Veterinarias	Google Scholar
40	Molecular characteristics and zoonotic potential of enteric protozoans in domestic small ruminants in Heilongjiang Province, Northeast China.	Hou, Liu, Zhou, Zhou, Zhang, Ma, Qiu, Wang, Gao	2025	Food and waterborne parasitology	PubMed

S/N	Title	Authors	Year	Journal	Search Source
41	Extracellular vesicles in intestinal protozoa: hidden mediators of host-parasite communication.	Fadaee, Lahouty, Ramzi, Alizadeh, Roshanfar, Kazemi, Spotin, Hatam-Nahavandi, Ahmadpour	2025	Gut pathogens	Scopus
42	Decoding Blastocystis-driven mechanisms in gut microbiota and host metabolism	Deng L, Tan KS	2025	Adv Sci (Weinh)	Web of Science
43	A cross - sectional investigation on the molecular infection rate and subtype distribution of	Sun, Tan, Zhang, Ren, Huang, Zhao	2025	Frontiers in cellular and infection microbiology	PubMed
44	Prevalence and subtype distribution of Blastocystis infections among community participants in Thailand: a systematic review and meta-analysis	Kotepui M, Popruk S, Kotepui KU, Masangkay FR, Wangdi K, Mahittikorn A, Stensvold CR	2025	Parasite	Scopus
45	Assessment of the Therapeutic Role of Allium tuncelianum Extract in Rats Infected with Blastocystis Subtype 3.	Aykur, Tosun G, ozgur	2025	Acta parasitologica	PubMed
46	Genetic characterization of enteric protozoan microorganisms in newly arrived migrants in Italy and correlation with the gut microbiome layout.	Marangi, Palladino, Valzano, Scicchitano, Turroni, Rampelli, Candela, Arena	2025	Travel medicine and infectious disease	Scopus
47	Atypical Presentation of Blastocystis-Related Dysentery in an HIV-Infected Patient	Candradikusuma, Kalim, Fitri, Pawestri, Winaris, Hayati, Putri, Rahmawati, Kaisar	2025	International medical case reports journal	Scopus
48	Molecular Identification and Genotyping of Blastocystis sp. in Patients with Gastrointestinal Symptoms in Kermanshah, Western Iran	Mirahmadi, Rahmati-Balaghaleh, Darabi, Zarean, Sharifi, Yousefnia, Etemadi, Parandin, Askari	2025	Archives of Razi Institute	Scopus

S/N	Title	Authors	Year	Journal	Search Source
49	Targeted pathogen profiling of ancient feces reveals common enteric infections in the Rio Zape Valley, 725-920 CE.	Capone, Holcomb, Lai, Meade, Reinhard, Brown	2025	PloS one	Web of Science
50	Suppression/competition PCR: A novel method to minimize unwanted amplicons in metabarcoding, with applications to parasite detection in fecal samples.	Carpani, Barta, Guy	2025	Journal of microbiological methods	PubMed
51	Exploring bacterial and eukaryotic communities in the gut microbiota of urban and rural cats (Felis catus) in Colombia.	Authors: Paez-Triana, L., Luna, N., Ramirez, J. D., Camargo, G., Reina, L. S., Cardona, G., Velandia, M. D., Zuniga, J. S., Patino, L. H., Ramirez, J. D., & Munoz, M	2025	Veterinary research communications	Web of Science
52	Prevalence of Intestinal Protozoa Among Patients Living with HIV in the Peruvian Amazon.	Otero-Rodriguez, Pinedo-Cancino, Casapia-Morales, Villacorta-Pezo, Mego-Campos, Parraguez-de-la-Cruz, Merino, Clark, Ramos-Rincon	2025	Tropical medicine and infectious disease	Scopus
53	Multiple Blastocystis subtypes in cetaceans from the Mediterranean Sea: Evidence of zoonotic potential and host-parasite interactions	Palomba, Rodriguez-Fernandez, Aco-Alburqueque, Carrus, Marcer, Marchiori, Santoro, Castrignano, Canestrelli, Mattiucci	2025	Food and waterborne parasitology	Scopus
54	Impact of Intestinal Parasitic Infections on Gut Epithelial Barrier and Inflammation among Foreign-Born Persons Living with HIV.	Reimer-McAtee, Serpa, McAtee, Ortega, Somasunderam, Arduino, Mejia, Utay	2025	The American journal of tropical medicine and hygiene	Web of Science

S/N	Title	Authors	Year	Journal	Search Source
55	Blastocystis infection in Tibetan antelopes (Pantholops hodgsonii) alters gut microbiota composition and function	Liu, Qin, Lei, Ma, Xie, Liu, Li, Ni, Yu, Liang, Shi, Qin, Jiang, Yan, Chen, Li, Sun	2025	Frontiers in cellular and infection microbiology	PubMed
56	Comparison of the Intestinal Microbiota of Patients with Urticaria and Healthy Controls: The Role of Blastocystis.	Ciftci, Macin, Kurtipek S, Arslan	2025	Pathogens (Basel, Switzerland)	PubMed
57	Commensal, pathogen, or passenger? Rethinking the role of	Heneberg	2025	One health (Amsterdam, Netherlands)	Scopus
58	The interplay between Blastocystis and human gut microbiota.	Lepczynska	2025	Annals of parasitology	Scopus
59	Frequency of Intestinal Protozoa in Patients Receiving Treatment at Van Special Physioactive Special Education and Rehabilitation Center.	Saygin, Aydemir, Ekici, Yilmaz	2025	Turkiye parazitolojii dergisi	PubMed
60	Blastocystis colonization among inland and forest periphery Negritos in Malaysia: The crucial role of sanitation and hygiene practices.	Rozani, Aazmi, Attah, Termizi M, Shahrizal, Idorus, Muslim	2025	Parasitology international	Web of Science
61	Blastocystis hominis infection in Southeast Asia: a systematic review of prevalence and risk factors	Riswana FR, Fitri LE, Winaris N	2025	Microbes Infect Dis	PubMed
62	Molecular characterization and zoonotic potential of Blastocystis subtypes in domestic pigs and cattle from Hainan, a tropical island province in China.	Zhang, Li, Lai, Wang, Li, Ren, Yu, Li, Liu, Qiang, Li, Zhou, Lei, Wu, Zhao, Lu	2025	Parasite (Paris, France)	PubMed
63	Prevalence and subtype distribution of Blastocystis infections among community participants in Thailand: a systematic review and meta-analysis	Kotepui M, Popruk S, Kotepui K, Masangkay F	2025	Parasite	Web of Science

S/N	Title	Authors	Year	Journal	Search Source
64	Crossing the species barrier: Bidirectional transmission of Blastocystis between pets and their owners	Heneberg P	2025	One Health	Web of Science
65	Molecular identification and genotyping of Blastocystis spp. in children with clinical symptoms in southeast Iran using PCR-sequencing method	Saryazdi, S. K., Kamyabi, H., & Bamorovat, M	2024	archives of razi institute	Web of Science
66	Cats and dogs as hosts of Blastocystis - what is the evidence?	Popruk S, Koompapong K, Mahittikorn A, Andersen LO, Stensvold CR	2024	Veterinary Parasitology	Web of Science
67	Global prevalence and subtype distribution of Blastocystis sp. in rodent populations: A systematic review and meta-analysis	Farzam, A., Karampour, A., Nazem-Sadati, S., Sadat-Madani, P., & Asghari, A	2024	Veterinary medicine and science	PubMed
68	Blastocystis colonization and associations with population parameters in Thai adults	Jinatham V, Yowang A, Stensvold CR, Michalopoulou E, Vichaslip T, Suwannahitatorn P, Popluechai S, Tsaousis AD, Gentekaki E	2024	PLOS Neglected Tropical Diseases	Scopus
69	Fecal microbiota transplantation from protozoa-exposed donors downregulates immune response in a germ-free mouse model, its role in immune response and physiology of the intestine	Partida-Rodriguez, O., Brown, E. M., Woodward, S. E., Cirstea, M., Reynolds, L. A., Petersen, C., Vogt, S. L., Pena-Diaz, J., Thorson, L., Arrieta, M. C., Hernandez, E. G., Rojas-Velazquez, L., Moran, P., Rivas, E. G., Serrano-Vazquez, A., Perez-Juarez, H., Torres, J., Ximenez, C., & Finlay, B. B	2024	PLOS ONE	PubMed
70	Epidemiological survey on prevalence and subtypes distribution of Blastocystis sp. in Southern Guizhou, China	Fu X, Lyu J, Shi Y, Cao B, Liu D, Yang X, Lin H, Liang Q, Liao D, He S	2024	Biomolecules & biomedicine	PubMed
71	[Genetic Diversity of Blastocystis in Diarrheal Cases: Identification of Subtypes and Alleles].	Tileklioglu E, Ertabaklar H	2024	Mikrobiyoloji Bulteni	PubMed

S/N	Title	Authors	Year	Journal	Search Source
72	Integrated Omics Reveal the Pathogenic Potential of Blastocystis sp. ST2	Cao M, Zhang S, Nan H, Huang J, Zhang C, Sun Y, Liu L, Wang Y, Lu X, Ma L	2024	Transboundary and Emerging Diseases	Web of Science
73	Blastocystis colonization and associations with population parameters in Thai adults	Jinatham V, Yowang A, Stensvold CR, Michalopoulou E, Vichaslip T, Suwannahitatorn P, Popluechai S, Tsaousis AD, Gentekaki E	2024	PLOS Neglected Tropical Diseases	Google Scholar
74	Unveiling Blastocystis epidemiology in Morocco: subtype diversity among clinical patients with and without gastrointestinal manifestations in the Meknes region	Boutahar M, Belaouni M, Ibrahimi A, Eljaoudi R, Aanniz T, Errami M, Koster PC, Dashti A, Carmena D	2024	Parasitology Research	PubMed
75	Subtype Distribution of Blastocystis spp. in Patients with Gastrointestinal Symptoms in Northern Spain	Matovelle C, Quilez J, Tejedor MT, Beltran A, Chueca P, Monteagudo LV	2024	Microorganisms	Scopus
76	Blastocystis: A Mysterious Member of the Gut Microbiome	Aykur M, Malatyali E, Demirel F, Comert-Kocak B, Gentekaki E, Tsaousis AD, Dogruman-Al F	2024	Microorganisms	PubMed
77	Rare occurrence of Blastocystis in sea turtles and insects (cockroaches, houseflies, and crickets) from several states in Peninsular Malaysia.	Hafiz M, Attah, Salleh M, Rusli, Haziqah F	2024	Tropical biomedicine	Web of Science
78	Epidemiological study of the intestinal parasite Blastocystis in Central Vietnam	Nguyen, L. D. N., Gantois, N., Hoang, T. T., Thi, B., Desramaut, J., Naguib, D., Tran, T. N., Truong, A. D., Even, G., Certad, G., Chabe, M	2024	Vietnam Journal of Endocrinology and Diabetes	Web of Science
79	Prevalence and molecular identification of protozoan intestinal parasitic infections in cancer patients and a control group.	Bahadorizadeh, Khanaliha, Ghorbandoust, Bokharei-Salim, Minaeian, Khodakarim, Ghalamkari, Salemi	2024	BMC infectious diseases	Scopus

S/N	Title	Authors	Year	Journal	Search Source
80	Enhancing enteric pathogen detection: implementation and impact of multiplex PCR for improved diagnosis and surveillance.	Mohtar, Mallah, Mardirossian, El-Bikai, Jisr, Soussi, Naoufal, Alam, Chaar	2024	BMC infectious diseases	Scopus
81	Epidemiological survey on prevalence and subtypes distribution of Blastocystis sp. in Southern Guizhou, China	Fu X, Lyu J, Shi Y, Cao B, Liu D, Yang X, Lin H, Liang Q, Liao D, He S	2024	Biomolecules & biomedicine	Scopus
82	A Worldwide Systematic Review and Meta-Analysis of the Prevalence and Subtype Distribution of Blastocystis sp. in Water Sources: A Public Health Concern	Mahdavi F, Maleki F, Mohammadi M	2024	Foodborne Pathog Dis	Web of Science
83	Blastocystis colonization and associations with population parameters in Thai adults	Jinatham V, Yowang A, Stensvold CR, Michalopoulou E, Vichaslip T, Suwannahitatorn P, Popluechai S, Tsaousis AD, Gentekaki E	2024	PLOS Neglected Tropical Diseases	Google Scholar
84	Occurrence and genotypic identification of Blastocystis sp., Enterocytozoon bieneusi, and Giardia duodenalis in dairy cattle in Heilongjiang Province, China.	Duan J, Fu Y, Lang J, Wang C, Zhang LYA	2024	Parasitology International	Web of Science
85	Integrated Omics Reveal the Pathogenic Potential of Blastocystis sp. ST2	Cao M, Zhang S, Nan H, Huang J, Zhang C, Sun Y, Liu L, Wang Y, Lu X, Ma L	2024	Transboundary and Emerging Diseases	Web of Science
86	Enhancing enteric pathogen detection: implementation and impact of multiplex PCR for improved diagnosis and surveillance.	Mohtar, Mallah, Mardirossian, El-Bikai, Jisr, Soussi, Naoufal, Alam, Chaar	2024	BMC infectious diseases	Web of Science

S/N	Title	Authors	Year	Journal	Search Source
87	Blastocystis occurrence and subtype diversity in European wild boar (Sus scrofa) from the Iberian Peninsula	Koster, P. C., Figueiredo, A. M., Maloney, J. G., Dashti, A., Bailo, B., Torres, R. T., Fonseca, C., Mysterud, A., Habela, M. A., Rivero-Juarez, A., Vicente, J., Serrano, E., Arnal, M. C., Fernandez de Luco, D., Armenteros, J. A., Balseiro, A., Cardona, G. A., Carvalho, J., Hipolito, D., Carmena, D	2024	Veterinary research	Web of Science
88	Molecular prevalence and subtype distribution of Blastocystis spp. among children who have diarrheia or are asymptomatic in Wenzhou, Zhejiang Province, China	Zhao W, Ren G, Wang L, Xie L, Wang J, Mao J, Sun Y, Lu G, Huang H	2024	Parasite	Web of Science
89	Molecular Prevalence and Subtypes Distribution of Blastocystis spp. in Humans of Latin America: A Systematic Review	Authors: Fusaro, C., Bernal, J. E., Baldiris-Avila, R., Gonzalez-Cuello, R., Cisneros-Lorduy, J., Reales-Ruiz, A., Castro-Orozco, R., & Sarria-Guzman, Y	2024	Tropical Medicine and Infectious Disease	Scopus
90	Occurrence and genotypic identification of Blastocystis sp., Enterocytozoon bieneusi, and Giardia duodenalis in dairy cattle in Heilongjiang Province, China.	Duan J, Fu Y, Lang J, Wang C, Zhang LYA	2024	Parasitology International	PubMed
91	Chronic urticaria associated with Blastocystis hominis infection.	Jafari, Bahrami, Nasiri-Kalmarzi, Abdoli	2024	Archives of dermatological research	PubMed
92	Clinical Care of Blastocystosis	Centers for Disease Control and Prevention	2024	CDC Resource	PubMed

S/N	Title	Authors	Year	Journal	Search Source
93	Clustering of Gastrointestinal Microorganisms in Human Stool Samples from Ghana.	Backhaus, S., Kann, S., Hahn, A., Weinreich, F., Blohm, S., Tanida, K., Feldt, T., Sarfo, F. S., Cristanziano, V. D., Loderstadt, U., Ehrhardt, J., Schoppen, S., Tagbor, H. K., Frickmann, H., & Eberhardt, K. A	2024	Pathogens (Basel, Switzerland)	Google Scholar
94	Molecular Prevalence of Blastocystis sp. from Patients with Diarrhea in the Republic of Korea	Ju J, Lee M	2024	Microorganisms	PubMed
95	Immune-mediated Bowel Disease: Role of Intestinal Parasites and Gut Microbiome	Chowdhury SR, Dey A, Gautam MK, Mondal S, Pawar S, Ranade A, Bora M, Gangwar M, Teli A, Mondal NS	2024	Current Pharmaceutical Design	PubMed
96	Anti-blastocystis activity of hop extracts in vitro	Pokhil SI, Kazmirchuk V, Tymchenko O, Yevsiukova VY, Melnyk A	2024	Infection, Inflammation and Immunity	Web of Science
97	Irritable Bowel Syndrome Associated with Blastocystis hominis or Without Relationship to It? A Case-Control Study and Minireview.	Maghsood, Kayedimajd, Motavallihaghi, Abedian, Kordi, Davoodi, Faizi, Soleymani	2024	Acta parasitologica	Google Scholar
98	Molecular prevalence and subtype distribution of Blastocystis spp. among children who have diarrheia or are asymptomatic in Wenzhou, Zhejiang Province, China	Zhao W, Ren G, Wang L, Xie L, Wang J, Mao J, Sun Y, Lu G, Huang H	2024	Parasite	Scopus
99	Occurrence and Subtype Distribution of Blastocystis in Smallholder Dairy Cattle in Bangladesh	Karim MR, Harun AB, Rehena J, Siddiki SHMF	2024	Journal of science and technology research	Google Scholar

S/N	Title	Authors	Year	Journal	Search Source
100	First molecular characterization of Blastocystis subtypes from domestic animals (sheep and cattle) and their animal-keepers in Ilam, western Iran: A zoonotic concern.	Shams M, Bahrami AM, Mousivand A, Shamsi L, Asghari A, Shahabi S, Sadrebazzaz A	2024	Journal of Eukaryotic Microbiology	Web of Science
101	dentification of Cryptosporidium parvum and Blastocystis hominis subtype ST3 in Cholga mussel and treated sewage: Preliminary evidence of fecal contamination in harvesting area	Suarez P, Vallejos-Almirall A, Fernandez I	2024	ood and Waterborne Parasitology	PubMed
102	Intestinal microbiota analysis of different Blastocystis subtypes and Blastocystis-negative individuals in Taiwan.	Huang, Yeh, Chiu, Huang, Chu, Huang, Cheng, Chen, Lin, Shih, Lin, Huang	2024	Biomedical journal	Google Scholar
103	An Update on Blastocystis: Possible Mechanisms of Blastocystis-Mediated Colorectal Cancer	Tocci S, Das S, Sayed IM	2024	Microorganisms	Google Scholar
104	Global prevalence and subtype distribution of Blastocystis sp. in rodent populations: A systematic review and meta-analysis	Farzam, A., Karampour, A., Nazem-Sadati, S., Sadat-Madani, P., & Asghari, A	2024	Veterinary medicine and science	PubMed
105	Fecal microbiota transplantation from protozoa-exposed donors downregulates immune response in a germ-free mouse model, its role in immune response and physiology of the intestine	Partida-Rodriguez, O., Brown, E. M., Woodward, S. E., Cirstea, M., Reynolds, L. A., Petersen, C., Vogt, S. L., Pena-Diaz, J., Thorson, L., Arrieta, M. C., Hernandez, E. G., Rojas-Velazquez, L., Moran, P., Rivas, E. G., Serrano-Vazquez, A., Perez-Juarez, H., Torres, J., Ximenez, C., & Finlay, B. B	2024	PLOS ONE	PubMed
106	Rare occurrence of Blastocystis in sea turtles and insects (cockroaches, houseflies, and crickets) from several states in Peninsular Malaysia.	Hafiz M, Attah, Salleh M, Rusli, Haziqah F	2024	Tropical biomedicine	Scopus

S/N	Title	Authors	Year	Journal	Search Source
107	Global prevalence and subtype distribution of Blastocystis sp. in rodent populations: A systematic review and meta-analysis	Farzam, A., Karampour, A., Nazem-Sadati, S., Sadat-Madani, P., & Asghari, A	2024	Veterinary medicine and science	PubMed
108	Molecular Prevalence of Blastocystis sp. from Patients with Diarrhea in the Republic of Korea	Ju J, Lee M	2024	Microorganisms	Google Scholar
109	Blastocystis hominis as a cause of chronic diarrhea in low-resource settings: A systematic review	Amoak S, Soldera J	2024	World journal of meta-analysis	PubMed
110	Occurrence and ultrastructural surface of Blastocystis isolated from water sources in Kedah and Penang, Malaysia.	Attah, Ong, Sanggari, Lee, Him N, Ismail, Haziqah F	2024	Tropical biomedicine	Scopus
111	First molecular characterization of Blastocystis subtypes from domestic animals (sheep and cattle) and their animal-keepers in Ilam, western Iran: A zoonotic concern.	Shams M, Bahrami AM, Mousivand A, Shamsi L, Asghari A, Shahabi S, Sadrebazzaz A	2024	Journal of Eukaryotic Microbiology	PubMed
112	Epidemiological survey on prevalence and subtypes distribution of Blastocystis sp. in Southern Guizhou, China	Fu X, Lyu J, Shi Y, Cao B, Liu D, Yang X, Lin H, Liang Q, Liao D, He S	2024	Biomolecules & biomedicine	PubMed
113	Occurrence and Genotypic Identification of Blastocystis Sp., Enterocytozoon Bieneusi, and Giardia Duodenalis in Dairy Cattle in Heilongjiang Province, China	Duan J, Qin H, Sun M, Fu Y, Lang J, Zhang A, Qin Z, Z G, Xu H, Li X, Wang C, Zhang L	2024	Veterinary Parasitology	Web of Science
114	Large-Scale Molecular Epidemiological Survey of Blastocystis sp. among Herbivores in Egypt and Assessment of Potential Zoonotic Risk	Naguib, D., Gantois, N., Desramaut, J., Dominguez, R., Arafat, N., Atwa, S., Even, G., Devos, D. P., Certad, G., Chabe, M., & Viscogliosi, E	2024	Microorganisms	Scopus
115	Genetic diversity and host specificity of Blastocystis in reptiles, Eastern Thailand.	Sutthikornchai, Abu, Pintong, Chiabchalard, Nokkaew, Toompong, Popruk, Stensvold	2024	Veterinary parasitology	Scopus

S/N	Title	Authors	Year	Journal	Search Source
116	Molecular Assessment of Blastocystis hominis in Patients with Diabetes Mellitus in Balad City, Saladin Province, Iraq	Ismaiel SK, Ali AA, Al-Assi AHA	2024	Medical Journal of Babylon	PubMed
117	Molecular Prevalence of Blastocystis sp. from Patients with Diarrhea in the Republic of Korea	Ju J, Lee M	2024	Microorganisms	Scopus
118	Molecular investigation of Blastocystis sp. infections in wild rodents from the Inner Mongolian Autonomous Region and Liaoning province, China: High prevalence and dominance of ST4	Liu L, Liu L, Liu L, Liu L, Liu L	2024	Parasite	Scopus
119	Colonization with two different Blastocystis subtypes in DSS-induced colitis mice is associated with strikingly different microbiome and pathological features	Deng L, Wojciech L, Png CW, Kioh DYQ, Gu Y, Aung TT, Malleret B, Chan ECY, Peng G, Zhang Y, Gascoigne NRJ, Tan KSW	2023	Theranostics	PubMed
120	Efficacy of Nitazoxanide, Nitazoxanide-Garlic Combination and Tinidazole in Treatment of Giardia duodenalis and Blastocystis hominis: Coproscopic Assessment	Abdo SM, Ghallab MMI, Zaghloul MS, Elhadad H	2023	Iranian Journal of Parasitology	PubMed
121	Progress of researches on Blastocystis hominis infection among patients with inflammatory bowel disease and irritable bowel syndrome	Li, Cai, Yu, Chen, Tian	2023	Chinese Journal of Schistosomiasis Control	Scopus
122	Comparison of Various Diagnostic Techniques for the Detection of Blastocystis Spp. and its Molecular Characterisation in Farm Animals in the United Arab Emirates	ElBakri A, Kanu GA, Salahat D, Hussein NM, Ibrahim ZA, Hasan HT, AbuOdeh R	2023	Journal of Veterinary Research	PubMed

S/N	Title	Authors	Year	Journal	Search Source
123	The application of 3base technology to diagnose eight of the most clinically important gastrointestinal protozoan infections	Aghazadeh, Jones, Perera, Nair, Tan, Clark, Curtis, Jones, Ellem, Olma, Stark, Melki, Coulston, Baker, Millar	2023	International journal of molecular sciences	Scopus
124	Molecular epidemiology and subtyping of Blastocystis sp. and its subtypes in celiac patients; a case control study	Jevinani, S. S., Rahimi, H. M., Asri, N., Rostami-Nejad, M., Ahmadipour, S., & Mirjalali, H	2023	Microbial Pathogenesis	PubMed
125	Site-specific incidence rate of Blastocystis hominis and its association with childhood malnutrition: findings from a multi-country birth cohort study	Haque MA, Gazi MA, Fahim SM, Faruque ASG, Mahfuz M, Ahmed T	2023	American Journal of Tropical Medicine and Hygiene	Google Scholar
126	Systematic Review and Meta-Analysis: Epidemiology of Human Blastocystis spp. Infection in Malaysia	Kumarasamy V, Rajamanikam A, Anbazhagan D, Atroosh WM, Azzani M, Subramaniyan V, Abdullah SR	2023	Tropical Medicine and Infectious Disease	Scopus
127	First report of Blastocystis subtype ST25 in calves in Turkey.	celik BA	2023	Polish Journal of Veterinary Sciences	Scopus
128	Subtype Distribution of Blastocystis in Turkiye.	Malatyali E, Ertabaklar H, Ertug S	2023	Turkish Journal of Parasitology	PubMed
129	Molecular Identification of Blastocystis hominis Isolates in Patients with Autoimmune Diseases	Mahmoud AM, Ismail KA, Khalifa OM, Abdel-Wahab MM, Hagag HM, Mahmoud MK	2023	Applied microbiology	Scopus

S/N	Title	Authors	Year	Journal	Search Source
130	Etiology and risk factors for diarrheal disease amongst rural and peri-urban populations in Cambodia, 2012-2018.	Kelly, Rachmat, Hontz, Sklar, Tran, Supaprom, Luy, Lina, Gregory, Sopheab, Brooks, Sutherland, Corson, Letizia	2023	PloS one	Web of Science
131	Biochemical and Microbiological Interactions of Molecularly Detected Blastocystis hominis: a cross-sectional study.	El Saftawy, E. A., Ghallab, M. M. I., Haydara, T., Ibrahim, A., Atta, S. A., Gad Allah, R. A. M., Kamel, M. N., & Morsy, S. M	2023	Parasitologists United Journal	PubMed
132	Identification of Blastocystis sp. in school children from a rural Mexican village: subtypes and risk factors analysis	Pineda-Rodriguez S, Romero-Valdovinos M, Martinez-Hernandez F, Rendon-Franco E, Olivo-Diaz A, Maravilla P, Rodriguez-Bataz E	2023	Parasitology Research	Scopus
133	Blastocystis subtype 3 among adolescents with gastrointestinal symptoms in fayoum governorate, egypt	Mohamed FI, KHALIL K	2023	Journal of the Egyptian Society of Parasitology	Web of Science
134	The associated risk of Blastocystis infection in cancer: A case control study	Labania LAB, Zoughbor S, Ajab S, Olanda M, Shantour SNM, Rasbi ZA	2023	Frontiers in Oncology	PubMed
135	Subtype Distribution of Blastocystis in Turkiye.	Malatyali E, Ertabaklar H, Ertug S	2023	Turkish Journal of Parasitology	Scopus
136	Progress of researches on Blastocystis hominis infection among patients with inflammatory bowel disease and irritable bowel syndrome	Li, Cai, Yu, Chen, Tian	2023	Chinese Journal of Schistosomiasis Control	Scopus
137	Epidemiological and Biochemical changes of patients infected with Blastocystis hominis and Irritable bowel syndrome	Kadhim JA, Jasim HS	2023	Tikrit Journal of Pure Science	PubMed

S/N	Title	Authors	Year	Journal	Search Source
138	Nitazoxanide: A Drug Repositioning Compound with Potential Use in Chagas Disease in a Murine Model	Arce-Fonseca M, Gutierrez-Ocejo RA, Rosales-Encina JL, Aranda-Fraustro A, Cabrera-Mata JJ, Rodriguez-Morales O	2023	Pharmaceuticals	PubMed
139	Comparison of Various Diagnostic Techniques for the Detection of Blastocystis Spp. and its Molecular Characterisation in Farm Animals in the United Arab Emirates	ElBakri A, Kanu GA, Salahat D, Hussein NM, Ibrahim ZA, Hasan HT, AbuOdeh R	2023	Journal of Veterinary Research	Scopus
140	Exploring Blastocystis genetic diversity in rural schoolchildren from Colombia using next-generation amplicon sequencing reveals significant associations between contact with animals and infection risk	Hernandez PC, Maloney JG, Molokin A, George NS, Morales L, Chaparro-Olaya J, Santin M	2023	Parasitology Research	Web of Science
141	Profiling of the fecal microbiota and circulating microRNA-16 in IBS subjects with Blastocystis infection : a case-control study	Olyaiee A, Yadegar A, Mirsamadi ES, Sadeghi A, Mirjalali H	2023	European Journal of Medical Research	PubMed
142	Molecular identification of blastocystis sp. in long-tailed macaque at alas purwo park	Kristijanto EY, Yuniarti WM, Mufasirin M, Hastutiek P, Suprihati E, Setiawan B, Wahyuningtyas PA, Mahendra D	2023	Buletin Veteriner Udayana	PubMed
143	Comparison of Various Diagnostic Techniques for the Detection of Blastocystis Spp. and its Molecular Characterisation in Farm Animals in the United Arab Emirates	ElBakri A, Kanu GA, Salahat D, Hussein NM, Ibrahim ZA, Hasan HT, AbuOdeh R	2023	Journal of Veterinary Research	Scopus
144	Profiling of the fecal microbiota and circulating microRNA-16 in IBS subjects with Blastocystis infection : a case-control study	Olyaiee A, Yadegar A, Mirsamadi ES, Sadeghi A, Mirjalali H	2023	European Journal of Medical Research	Web of Science

S/N	Title	Authors	Year	Journal	Search Source
145	Prevalence, potential risk factors and genetic diversity of Blastocystis in ruminant livestock animals from Penang, Malaysia.	Rauff-Adedotun, Lee, Talib A, Shaari, Yahaya, Termizi M	2023	Parasitology research	Scopus
146	Circulation and colonisation of Blastocystis subtypes in schoolchildren of various ethnicities in rural northern Thailand	Pitaksakulrat, O., Srisawat, N., Thanchomnang, T., Saneerot, P., Phosiri, K., & Maleewong, W	2023	Epidemiology and Infection	Scopus
147	Colonization with ubiquitous protist Blastocystis ST1 ameliorates DSS-induced colitis and promotes beneficial microbiota and immune outcomes	Deng L, Wojciech L, Png CW, Chan ECY, Zhang Y, Gascoigne NRJ, Tan KSW	2023	npj biofilms and microbiomes	PubMed
148	Frequency, Associated Factors, Clinical Symptoms, and Subtypes of Blastocystis sp. in Patients With Irritable Bowel Syndrome and Healthy Individuals: A Case-Control Study	Norouzi M, Pirestani M, Arefian E, Dalimi A, Sadraei J, Mirjalali H, Olyaiee A	2023	International Journal of Enteric Pathogens	Google Scholar
149	Spread of Intestinal Parasites in Patients Presenting with Gastrointestinal Complaints.	Ekici, Gunay, Sahin, Aydemir, Yilmaz	2023	Turkiye parazitolojii dergisi	PubMed
150	Circulation and colonisation of Blastocystis subtypes in schoolchildren of various ethnicities in rural northern Thailand	McCain AH, Gruneck L, Popluechai S, Tsaousis AD, Gentekaki E	2023	Epidemiology and Infection	Web of Science
151	Molecular identification of blastocystis sp. in long-tailed macaque at alas purwo park	Kristijanto EY, Yuniarti WM, Mufasirin M, Hastutiek P, Suprihati E, Setiawan B, Wahyuningtyas PA, Mahendra D	2023	Buletin Veteriner Udayana	Web of Science
152	Detection of Blastocystis Hominis by Method of Cultivation in The Feces of Orphanage Children in Pekanbaru, Riau Province, Indonesia	Maryanti E, Lesmana SD, Mislindawati M, Siagian FE	2023	Journal of biomedicine and translational research	Scopus

S/N	Title	Authors	Year	Journal	Search Source
153	Blastocystis occurrence in water sources worldwide from 2005 to 2022: a review.	Attah, Sanggari, Li, Him N, Ismail, Termizi M	2023	Parasitology research	Web of Science
154	Circulation and colonisation of Blastocystis subtypes in schoolchildren of various ethnicities in rural northern Thailand - Erratum.	McCain, Gruneck, Popluechai, Tsaousis, Gentekaki	2023	Epidemiology and infection	Scopus
155	Blastocystis subtypes in raw vegetables from street markets in northern Thailand.	Jinatham, Wandee, Nonebudsri, Popluechai, Tsaousis, Gentekaki	2023	Parasitology research	Scopus
156	Prevalence and associated risk factors of intestinal parasites among schoolchildren in Ecuador, with emphasis on the molecular diversity of Giardia duodenalis, Blastocystis sp. and Enterocytozoon bieneusi	Tapia-Veloz EC, Gozalbo MER, Guillen M, Dashti A, Bailo B, Koster PC, Santin M, Carmena D, Trelis M	2023	PLOS Neglected Tropical Diseases	PubMed
157	Prevalence and associated risk factors of intestinal parasites among schoolchildren in Ecuador, with emphasis on the molecular diversity of Giardia duodenalis, Blastocystis sp. and Enterocytozoon bieneusi	Tapia-Veloz EC, Gozalbo MER, Guillen M, Dashti A, Bailo B, Koster PC, Santin M, Carmena D, Trelis M	2023	PLOS Neglected Tropical Diseases	Web of Science
158	Prevalence, potential risk factors and genetic diversity of Blastocystis in ruminant livestock animals from Penang, Malaysia.	Rauff-Adedotun, Lee, Talib A, Shaari, Yahaya, Termizi M	2023	Parasitology research	Scopus
159	Spread of Intestinal Parasites in Patients Presenting with Gastrointestinal Complaints.	Ekici, Gunay, Sahin, Aydemir, Yilmaz	2023	Turkiye parazitolojii dergisi	PubMed
160	Epidemiology of Blastocystis Infection: A Review of Data from Poland in Relation to Other Reports	Rudzinska M, Sikorska K	2023	Pathogens	Scopus
161	Circulation and colonisation of Blastocystis subtypes in schoolchildren of various ethnicities in rural northern Thailand	McCain AH, Gruneck L, Popluechai S, Tsaousis AD, Gentekaki E	2023	Epidemiology and Infection	Scopus

S/N	Title	Authors	Year	Journal	Search Source
162	Division of Blastocystis ST10 into three new subtypes: ST42-ST44.	Santin M, Figueiredo A, Molokin A, George NS, Koster PC, Dashti A, Gonzalez-Barrio D, Carmena D, Maloney JG	2023	Journal of Eukaryotic Microbiology	Web of Science
163	First Epidemiological Survey on the Prevalence and Subtypes Distribution of the Enteric Parasite Blastocystis sp. in Vietnam	Nguyen, L. D. N., Gantois, N., Hoang, T. T., Do, B. T., Desramaut, J., Naguib, D., Tran, T. N., Truong, A. D., Even, G., Certad, G., Chabe, M., & Viscogliosi, E	2023	Microorganisms	PubMed
164	Prevalence of Blastocystis sp. among cooks in the region of Fez-Meknes (Morocco)	Boutahar M, Er-Rami M, Belaouni M	2023	Helminthologia	Scopus
165	Exploring Blastocystis genetic diversity in rural schoolchildren from Colombia using next-generation amplicon sequencing reveals significant associations between contact with animals and infection risk	Hernandez PC, Maloney JG, Molokin A, George NS, Morales L, Chaparro-Olaya J, Santin M	2023	Parasitology Research	Web of Science
166	Blastocystis subtypes in raw vegetables from street markets in northern Thailand.	Jinatham, Wandee, Nonebudsri, Popluechai, Tsaousis, Gentekaki	2023	Parasitology research	PubMed
167	Metataxomics reveals Blastocystis subtypes mixed infections in Colombian children	Garcia-Montoya GM, Galvan-Diaz AL, Alzate JF	2023	Infection, Genetics and Evolution	Scopus
168	Molecular Epidemiology and Genetic Diversity of the Enteric Protozoan Parasite Blastocystis sp. in the Northern Egypt Population	Naguib DM, Gantois N, Desramaut J, Arafat N, Mandour M, Abdelmaogood AKK, Mosa AF, Denoyelle C, Even G, Certad G, Chabe M, Viscogliosi E	2023	Pathogens	PubMed

S/N	Title	Authors	Year	Journal	Search Source
169	Mobilization of Natural Substances Against Blastocystis sp. With a Focus on Medicinal Plants: A Mini-Review	Ghazi Z, Pirestani M	2023	International Journal of Enteric Pathogens	Web of Science
170	Prevalence of Blastocystis sp. in Morocco: comparative assessment of three diagnostic methods and characterization of parasite forms in Jones' culture medium	Boutahar M, Belaouni M, Ibrahimi A, Eljaoudi R, Aanniz T, Er-Rami M	2023	Parasite	PubMed
171	Prevalence and distribution of subtypes of Blastocystis in Asiatic brush-tailed porcupines (Atherurus macrourus), bamboo rats (Rhizomys pruinosus), and masked palm civets (Paguma larvata) farmed in Hainan, China	Zhao W, Zhang Y, Li J, Ren G, Qiang Y, Wang Y, Lai X, Lei S, Liu R, Chen Y, Huang H, Li W, Lu G, Tan F	2023	Parasite	Scopus
172	Exploring Blastocystis genetic diversity in rural schoolchildren from Colombia using next-generation amplicon sequencing reveals significant associations between contact with animals and infection risk	Hernandez PC, Maloney JG, Molokin A, George NS, Morales L, Chaparro-Olaya J, Santin M	2023	Parasitology Research	Web of Science
173	Advances in the axenic isolation methods of Blastocystis sp. and their applications.	Mei X, Wei L, Su C, Yang Z, Tian X, Zhang Z, Wang S	2023	Parasitology	Web of Science
174	Gastrointestinal Pathogens in Multi-Infected Individuals: A Cluster Analysis of Interaction.	Backhaus, Frickmann, Hagen, Concha, Molitor, Hoerauf, Kann	2023	Microorganisms	Google Scholar
175	Mobilization of Natural Substances Against Blastocystis sp. With a Focus on Medicinal Plants: A Mini-Review	Ghazi Z, Pirestani M	2023	International Journal of Enteric Pathogens	PubMed

S/N	Title	Authors	Year	Journal	Search Source
176	Systematic Review and Meta-Analysis: Epidemiology of Human Blastocystis spp. Infection in Malaysia	Kumarasamy V, Rajamanikam A, Anbazhagan D, Atroosh WM, Azzani M, Subramaniyan V, Abdullah SR	2023	Tropical Medicine and Infectious Disease	Scopus
177	Exploring Blastocystis genetic diversity in rural schoolchildren from Colombia using next-generation amplicon sequencing reveals significant associations between contact with animals and infection risk	Hernandez PC, Maloney JG, Molokin A, George NS, Morales L, Chaparro-Olaya J, Santin M	2023	Parasitology Research	Google Scholar
178	Detection of Blastocystis Hominis by Method of Cultivation in The Feces of Orphanage Children in Pekanbaru, Riau Province, Indonesia	Maryanti E, Lesmana SD, Mislindawati M, Siagian FE	2023	Journal of biomedicine and translational research	Web of Science
179	Blastocystis occurrence in water sources worldwide from 2005 to 2022: a review.	Attah, Sanggari, Li, Him N, Ismail, Termizi M	2023	Parasitology research	Scopus
180	Site-specific incidence rate of Blastocystis hominis and its association with childhood malnutrition: findings from a multi-country birth cohort study	Haque MA, Gazi MA, Fahim SM, Faruque ASG, Mahfuz M, Ahmed T	2023	American Journal of Tropical Medicine and Hygiene	Scopus
181	Prevalence, potential risk factors and genetic diversity of Blastocystis in ruminant livestock animals from Penang, Malaysia.	Rauff-Adedotun, Lee, Talib A, Shaari, Yahaya, Termizi M	2023	Parasitology research	Google Scholar
182	Extensive prevalence and significant genetic differentiation of Blastocystis in high- and low-altitude populations of wild rhesus macaques in China	Yu M, Yao Y, Xiao H, Xie M, Xiong Y, Yang S, Ni Q, Zhang M, Xu H	2023	Parasites & Vectors	PubMed

S/N	Title	Authors	Year	Journal	Search Source
183	Subtypes and phylogenetic analysis of Blastocystis sp. isolates from West Ismailia, Egypt	Ahmed SA, El-Mahallawy HS, Mohamed SF, Angelici MC, Hasapis K, Saber T, Karanis P	2022	Dental science reports	Web of Science
184	The prevalence of Blastocystis sp. and its relationship with gastrointestinal disorders and risk factors	Viesy S, Rezaei Z, Pouladi I, Mirzaei A, Abdi J	2022	Iranian Journal of Parasitology	Web of Science
185	Experimental colonization with Blastocystis ST4 is associated with protective immune responses and modulation of gut microbiome in a DSS-induced colitis mouse model.	Deng L, Wojciech L, Png C, Koh E, Aung T	2022	Cellular and Molecular Life Sciences	PubMed
186	Intestinal immune responses to commensal and pathogenic protozoa	Sardinha-Silva A, Alves-Ferreira EVC, Grigg ME	2022	Frontiers in Immunology	Web of Science
187	Eficacia y tolerabilidad de Nitazoxanida para parasitismo intestinal en escolares atendidos en el Centro de Salud de la Universidad del Quindio	Castillo HAN, Cardona JAM, Ospina CN, Morales PCA, Marin JEG	2022	Revista medica de Risaralda	Web of Science
188	Distinct phenotypic variation of Blastocystis sp. ST3 from urban and Orang Asli population—An influential consideration during sample collection in surveys	Rajamanikam A, Hooi HS, Kudva M, Samudi C, Govind SK	2022	Biology	PubMed
189	Natural infection ofBlastocystis ST6 among commercial quails (Coturnix coturnix) in Penang, Malaysia.	Rauff-Adedotun, Douglas, Rajamanikam, Suriaty A, Haziqah F	2022	Tropical biomedicine	PubMed
190	Blastocystis	Gabrielli S, Rombach W, Jembrana JKK	2022	Encyclopedia of Infection and Immunity	Google Scholar
191	Blastocystis in tap water of a community in northern Thailand.	Jinatham, Nonebudsri, Wandee, Popluechai, Tsaousis, Gentekaki	2022	Parasitology international	Scopus

S/N	Title	Authors	Year	Journal	Search Source
192	Are Blastocystis hominis and Cryptosporidium spp. playing a positive role in colorectal cancer risk? A systematic review and meta-analysis	Taghipour A, Rayatdoost E, Bairami A, Bahadory S, Abdoli A	2022	Infectious Agents and Cancer	PubMed
193	Gut Microbiota Shifting in Irritable Bowel Syndrome: The Mysterious Role of Blastocystis sp.	Olyaiee A, Sadeghi A, Yadegar A, Mirsamadi ES, Mirjalali H	2022	Frontiers in Medicine	Scopus
194	Molecular Prevalence and Phylogenetic Characterization of Blastocystis in Cattle in Kayseri Province, Turkey	Tavur A, onder Z	2022	Kocatepe veteriner dergisi	Web of Science
195	Infection with pathogenic Blastocystis ST7 is associated with decreased bacterial diversity and altered gut microbiome profiles in diarrheal patients	Deng L, Lee JWJ, Tan KSW	2022	Parasites & Vectors	Google Scholar
196	Identification and Genetic Characterization of Blastocystis Species in Patients from Makkah, Saudi Arabia	Wakid MH, Aldahhasi WT, Alsulami MN, El-Kady AM, Elshabrawy HA	2022	Infection and Drug Resistance	Google Scholar
197	Blastocystis sp. Subtypes Colonisation and their Association with Clinical Diseases: A Systematic Review	Shaari, N. S. M., Abdullah, N. S. B., Lee, I. L., Omar, R., Sulaiman, W. S. W., & Nor, N. A. M	2022	ASM science journal	Web of Science
198	Gut Microbiota Shifting in Irritable Bowel Syndrome: The Mysterious Role of Blastocystis sp.	Olyaiee A, Sadeghi A, Yadegar A, Mirsamadi ES, Mirjalali H	2022	Frontiers in Medicine	Scopus
199	Identification and Molecular Characterization of Four New Blastocystis Subtypes Designated ST35-ST38	Maloney JG, Molokin A, Segui R, Maravilla P, Martinez-Hernandez F, Villalobos G, Tsaousis AD, Gentekaki E, Munoz-Antoli C, Klisiowicz DDR, Oishi CY, Toledo R, Esteban J, Koster PC, Lucio AD, Dashti A, Bailo B, Calero-Bernal R, Gonzalez-Barrio D, Carmena D, Santin M	2022	Microorganisms	PubMed

S/N	Title	Authors	Year	Journal	Search Source
200	Higher amoebic and metronidazole resistant forms of Blastocystis sp. seen in schizophrenic patients	Franklin F, Rajamanikam A, Raju CS, Gill JS, Francis B, Sy-Cherng LW, Kumar S	2022	Parasites & Vectors	PubMed
201	Experimental colonization with Blastocystis ST4 is associated with protective immune responses and modulation of gut microbiome in a DSS-induced colitis mouse model.	Deng L, Wojciech L, Png C, Koh E, Aung T	2022	Cellular and Molecular Life Sciences	Scopus
202	Blastocystis in captivated and free-ranging wild animals worldwide: a review.	Sanggari A, Komala T, Rauff-Adedotun AA, Awosolu OB, Attah O, Haziqah MTF	2022	Tropical Biomedicine	Scopus
203	Higher amoebic and metronidazole resistant forms of Blastocystis sp. seen in schizophrenic patients	Franklin F, Rajamanikam A, Raju CS, Gill JS, Francis B, Sy-Cherng LW, Kumar S	2022	Parasites & Vectors	Google Scholar
204	Blastocystis sp. Subtypes Colonisation and their Association with Clinical Diseases: A Systematic Review	Shaari, N. S. M., Abdullah, N. S. B., Lee, I. L., Omar, R., Sulaiman, W. S. W., & Nor, N. A. M	2022	ASM science journal	PubMed
205	Investigating the Effect of Silver, Chitosan, and Curcumin Nanoparticles on Blastocystis spp. and Comparing it With Metronidazole In Vitro	Sadraei J, Pirestani M, Bahadory S	2022	International Journal of Enteric Pathogens	Scopus
206	Should we treat Blastocystis sp.? A double-blind placebo-controlled randomized pilot trial.	Should we treat Blastocystis sp.? A double-blind placebo-controlled randomized pilot trial	2022	Clinical Infectious Diseases	PubMed

S/N	Title	Authors	Year	Journal	Search Source
207	Detection, Molecular Identification and Transmission of the Intestinal Protozoa Blastocystis sp. in Guinea from a Large-Scale Epidemiological Study Conducted in the Conakry Area	Guilavogui T, Gantois N, Even G, Desramaut J, Dautel E, Denoyelle C, Cisse FI, Toure SC, Kourouma BL, Sawant M, Chabe M, Certad G, Viscogliosi E	2022	Microorganisms	Web of Science
208	Experimental colonization with Blastocystis ST4 is associated with protective immune responses and modulation of gut microbiome in a DSS-induced colitis mouse model	Deng L, Wojciech L, Png CW, Koh EY, Aung TT, Kioh DYQ, Chan ECY, Malleret B, Zhang Y, Peng G, Gascoigne NRJ, Tan KSW	2022	Cellular and Molecular Life Sciences	Scopus
209	Molecular investigation of Blastocystis sp. and its subtypes in cancer patients under chemotherapy in Aegean region, Turkey	Oner, T., Karabey, M. S., Can, H., Doskaya, A. D., Karakavuk, M., Gul, A., Koseoglu, A. E., Doskaya, M., Can, C., Guruz, A. Y., Kaya, S., Pektas, B., & Gokmen, A. A	2022	Acta Tropica	Google Scholar
210	Medicinal plants as natural anti-parasitic agents against Blastocystis species.	El-Sayed NM, Masoud NG	2022	Recent advances in anti-infective drug discovery	Web of Science
211	Molecular subtyping of Blastocystis sp. detected in patients at a large tertiary referral hospital in Lusaka, Zambia	Munsaka G, Hayashida K, Mubemba B, Simulundu E, Mulunda NR, Pule R, Sianongo S, Makuluni M, Muleya W, Changula K, Chitanga S, Mutengo MM	2022	Frontiers in Parasitology	Scopus
212	Animal, Herd and Feed Characteristics Associated with Blastocystis Prevalence and Molecular Diversity in Dairy Cattle from the North of France	Audebert C, Gantois N, Ducrocq S, Darras M, Merlin S, Martel S, Viscogliosi E, Even G, Chabe M	2022	Parasitologia	PubMed

S/N	Title	Authors	Year	Journal	Search Source
213	Prevalence, Subtype Distribution and Zoonotic Significance of Blastocystis sp. Isolates from Poultry, Cattle and Pets in Northern Egypt	Naguib DM, Gantois N, Desramaut J, Arafat N, Even G, Certad G, Chabe M, Viscogliosi E	2022	Microorganisms	PubMed
214	Occurrence and subtyping of Blastocystis in coypus (Myocastor coypus) in China	Liu X, Ni F, Wang R, Li J, Ge Y, Yang X, Qiu M, Zhang L	2022	Parasites & Vectors	PubMed
215	The prevalence and subtype distribution of Blastocystis sp. in humans and domestic animals in family units in Heilongjiang Province, China	Wang, J., Zhao, W., Xie, H., Li, J., Guo, J., Song, J., & Zhang, L	2022	Acta Tropica	Web of Science
216	Molecular Epidemiology and Subtype Distributions of Blastocystis Sp. in Humans and Non-Human Primates	Zhang LYA, Wang L, Li J	2022	Global journal of epidemiology and public health	Scopus
217	Identification and Genetic Characterization of Blastocystis Species in Patients from Makkah, Saudi Arabia	Wakid MH, Aldahhasi WT, Alsulami MN, El-Kady AM, Elshabrawy HA	2022	Infection and Drug Resistance	Web of Science
218	Distribution, genetic diversity, and zoonotic significance of Blastocystis subtypes in pet dogs	Mahdavi F, Asghari A, Shahabi S, Shamsi L, Soltani-Jazi F, Sadrebazzaz A, Shams M	2022	Comp Immunol Microbiol Infect Dis	Scopus
219	Molecular Epidemiology of Blastocystis in Confined Slow Lorises, Macaques, and Gibbons	Ni Q, Dong S, Fan Y, Wan W, Teng P, Zhu S, Liang X, Xu H, Yao Y, Zhang M, Xie M	2022	Animals	Scopus
220	Should we treat Blastocystis sp.? A double-blind placebo-controlled randomized pilot trial.	Krogsgaard, L. R., Engsbro, A. L., Stensvold, C. R., Nielsen, J., Bytzer, P., & Petersen, A. M	2022	Clinical Infectious Diseases	Web of Science
221	An update on the distribution of Blastocystis subtypes in the Americas	Jimenez P, Munoz M, Ramirez JD	2022	Heliyon	Scopus

S/N	Title	Authors	Year	Journal	Search Source
222	Investigating the Effect of Silver, Chitosan, and Curcumin Nanoparticles on Blastocystis spp. and Comparing it With Metronidazole In Vitro	Sadraei J, Pirestani M, Bahadory S	2022	International Journal of Enteric Pathogens	Scopus
223	The regulatory function of Blastocystis spp. on the immune inflammatory response in the gut microbiome	Rojas-Velazquez L, Moran P, Serrano-Vazquez A, Portillo-Bobadilla T, Gonzalez E, Perez-Juarez H, Hernandez E, Partida-Rodriguez O, Nieves-Ramirez M, Padilla AM, Zaragoza M, Ximenez C	2022	Frontiers in Cellular and Infection Microbiology	Scopus
224	Experimental colonization with Blastocystis ST4 is associated with protective immune responses and modulation of gut microbiome in a DSS-induced colitis mouse model	Deng L, Wojciech L, Png C, Koh E, Aung T	2022	Cellular and Molecular Life Sciences	PubMed
225	Medicinal plants as natural anti-parasitic agents against Blastocystis species.	El-Sayed NM, Masoud NG	2022	Recent advances in anti-infective drug discovery	Scopus
226	Blastocystis sp. Carriage and Irritable Bowel Syndrome: Is the Association Already Established?	Salvador F, Lobo B, Goterris L, Alonso-Cotoner C, Santos J, Sulleiro E, Bailo B, Carmena D, Sanchez-Montalva A, Bosch-Nicolau P, Espinosa-Pereiro J, Fuentes I, Molina I	2021	Biology	PubMed
227	Molecular identification and subtype distribution of Blastocystis sp. in farm and pet animals in Turkey	Onder, Z., Yildirim, A., Pekmezci, D., Duzlu, O., Pekmezci, G. Z., Ciloglu, A., Simsek, E., Kokcu, N. D., Yetismis, G., Ercan, N., & Inci, A	2021	Acta Tropica	Web of Science

S/N	Title	Authors	Year	Journal	Search Source
228	Blastocystis sp. Prevalence and Subtypes Distribution amongst Syrian Refugee Communities Living in North Lebanon.	Khaled S, Khaled S, Gantois N, Ayoubi A, Even G, Sawant M, Houmayraa JE, Nabot M, Benamrouz-Vanneste S, Chabe M, Certad G, Safadi DE, Dabboussi F, Hamze M, Viscogliosi E	2021	Microorganisms	Google Scholar
229	Treatment strategies for Nitroimidazole-refractory giardiasis: A systematic review.	Bourque DL, Neumayr A, Neumayr A, Neumayr A, Libman M, Chen LH, Chen LH	2021	Journal of Travel Medicine	PubMed
230	Genetic characterization and zoonotic potential of Blastocystis from wild animals in Sichuan Wolong National Natural Reserve, Southwest China	Chen S, Meng W, Zhou Z, Deng L, Shi X, Chai Y, Liu H, Cheng Y, Zhong Z, Fu H, Shen L, Zhang K, He T, Peng G	2021	Parasite	Google Scholar
231	Blastocystis subtypes in patients with diabetes mellitus from the Midwest region of Brazil.	Melo, G. B. D., Mazzaro, M. C., Gomes-Gouvea, M. S., Santos, A. D., Souza, L. V. D., Elias-Oliveira, J., Gryschek, R. C. B., Rodrigues, R., & Paula, F. M. D	2021	Revista Do Instituto De Medicina Tropical De Sao Paulo	Google Scholar
232	Investigation of Isolated Blastocystis Subtypes from Cancer Patients in Turkey	Mulayim S, Aykur M, Dagci H, Dalkilic S, Aksoy A, Kaplan M	2021	Acta Parasitologica	Scopus
233	Occurrence and molecular characterization of Cryptosporidium spp., Giardia duodenalis, Enterocytozoon bieneusi, and Blastocystis sp. in captive wild animals in zoos in Henan, China.	Zhang K, Zheng S, Wang Y, Wang K, Wang Y, Gazizova A, Han K, Yu F, Chen Y, Zhang L	2021	BMC Veterinary Research	Google Scholar
234	Molecular Subtyping of Blastocystis sp. Isolated from Farmed Animals in Southern Italy.	Gabrielli S, Palomba M, Furzi F, Brianti E, Gaglio G, Napoli E, Rinaldi L, Alburqueque RA, Mattiucci S	2021	Microorganisms	Scopus

S/N	Title	Authors	Year	Journal	Search Source
235	Association of Blastocystis ST6 with higher protease activity among symptomatic subjects.	Karamati SA, Karamati SA, Mirjalali H, Niyyati M, Yadegar A, Aghdaei HA, Haghighi A, Tabaei SJS	2021	BMC Microbiology	Web of Science
236	Zoonotic Transmission of Blastocystis Subtype 1 among People in Eastern Communities of Thailand: Organic Fertilizer from Pig Feces as a Potential Source.	Ruang-areerate T, Piyaraj P, Suwannahitatorn P, Ruang-areerate P, Thita T, Naaglor T, Witee U, Sakboonyarat B, Leelayoova S, Mungthin M	2021	Microbiology spectrum	Web of Science
237	Occurrence and molecular characterization of Cryptosporidium spp., Giardia duodenalis, Enterocytozoon bieneusi, and Blastocystis sp. in captive wild animals in zoos in Henan, China.	Zhang K, Zheng S, Wang Y, Wang K, Wang Y, Gazizova A, Han K, Yu F, Chen Y, Zhang L	2021	BMC Veterinary Research	Scopus
238	Treatment strategies for Nitroimidazole-refractory giardiasis: A systematic review.	Bourque DL, Neumayr A, Neumayr A, Neumayr A, Libman M, Chen LH, Chen LH	2021	Journal of Travel Medicine	Scopus
239	Molecular detection of Cryptosporidium spp., Giardia duodenalis, and Enterocytozoon bieneusi in school children at the Thai-Myanmar border	Sutthikornchai C, Popruk S, Mahittikorn A, Arthan D, Soonthornworasiri N, Paratthakonkun C, Feng Y, Xiao L	2021	Parasitology Research	PubMed
240	Blastocystis Colonization Alters the Gut Microbiome and, in Some Cases, Promotes Faster Recovery From Induced Colitis.	Billy, V., Lhotská, Z., Jirků, M., Kadlecová, O., Frgelecová, L., Parfrey, L. W., & Pomajbíková, K	2021	Frontiers in Microbiology	Scopus
241	Blastocystis subtypes in patients with diabetes mellitus from the Midwest region of Brazil.	Melo, G. B. D., Mazzaro, M. C., Gomes-Gouvêa, M. S., Santos, A. D., Souza, L. V. D., Elias-Oliveira, J., Gryschek, R. C. B., Rodrigues, R., & Paula, F. M. D	2021	Revista Do Instituto De Medicina Tropical De Sao Paulo	Web of Science

S/N	Title	Authors	Year	Journal	Search Source
242	Identification of Multiple Blastocystis Subtypes in Domestic Animals From Colombia Using Amplicon-Based Next Generation Sequencing.	Higuera A, Herrera G, Jimenez P, Garcia-Corredor DJ, Pulido-Medellin MO, Bulla-Castaneda DM, Pinilla JC, Moreno-Perez DA, Maloney JG, Santin M, Ramirez JD	2021	Frontiers in Veterinary Science	Web of Science
243	Investigation of neglected protists Blastocystis sp. and Dientamoeba fragilis in immunocompetent and immunodeficient diarrheal patients using both conventional and molecular methods.	Sarzhanov F, Sarzhanov F, Dogruman-Al F, Santin M, Maloney JG, Gureser AS, Karasartova D, Taylan-Ozkan A, Taylan-Ozkan A	2021	PLOS Neglected Tropical Diseases	PubMed
244	Prevalence and molecular subtyping of Blastocystis in patients with Clostridium difficile infection, Singapore.	Deng L, Deng L, Tay H, Peng G, Lee JWJ, Lee JWJ, Tan KSW	2021	Parasites & Vectors	Scopus
245	Treatment strategies for Nitroimidazole-refractory giardiasis: A systematic review.	Bourque DL, Neumayr A, Neumayr A, Neumayr A, Libman M, Chen LH, Chen LH	2021	Journal of Travel Medicine	Web of Science
246	Prevalence and Subtype Analysis of Blastocystis hominis Isolated from Patients in the Northeast of Iran	Salehi M, Mardaneh J, Niazkar HR, Minooeianhaghighi M, Arshad E, Soleimani F, Mohammadzadeh A	2021	Journal of Parasitology Research	PubMed
247	Next-generation sequencing reveals wide genetic diversity of Blastocystis subtypes in chickens including potentially zoonotic subtypes	Maloney JG, Cunha MJRD, Cunha MJRD, Cunha MJRD, Molokin A, Cury MC, Santin M	2021	Parasitology Research	Scopus
248	Genetic characterization of Blastocystis from poultry, livestock animals and humans in the southwest region of Iran-Zoonotic implications.	Salehi R, Rostami A, Mirjalali H, Stensvold CR, Haghighi A	2021	Transboundary and Emerging Diseases	PubMed
249	Blastocystis in the faeces of children from six distant countries: prevalence, quantity, subtypes and the relation to the gut bacteriome.	Cinek O, Polackova K, Odeh R, Alassaf A, Kramna L, Ibekwe MAU, Majaliwa E, Ahmadov G, Elmahi BME, Mekki H, Oikarinen S, Lebl J, Abdullah MA	2021	Parasites & Vectors	Web of Science

S/N	Title	Authors	Year	Journal	Search Source
250	The neglected role of Blastocystis sp. and Giardia lamblia in development of irritable bowel syndrome: A systematic review and meta-analysis	Abedi SH, Fazlzadeh A, Mollalo A, Sartip B, Mahjour S, Bahadory S, Taghipour A, Rostami A	2021	Microbial Pathogenesis	PubMed
251	Molecular Characterization of Blastocystis from Animals and Their Caregivers at the Gdansk Zoo (Poland) and the Assessment of Zoonotic Transmission.	Rudzinska M, Kowalewska B, Waleron M, Kalicki M, Sikorska K, Szostakowska B	2021	Biology	Scopus
252	Identification of Multiple Blastocystis Subtypes in Domestic Animals From Colombia Using Amplicon-Based Next Generation Sequencing.	Higuera A, Herrera G, Jimenez P, Garcia-Corredor DJ, Pulido-Medellin MO, Bulla-Castaneda DM, Pinilla JC, Moreno-Perez DA, Maloney JG, Santin M, Ramirez JD	2021	Frontiers in Veterinary Science	Google Scholar
253	Molecular detection of Cryptosporidium spp., Giardia duodenalis, and Enterocytozoon bieneusi in school children at the Thai-Myanmar border	Sutthikornchai C, Popruk S, Mahittikorn A, Arthan D, Soonthornworasiri N, Paratthakonkun C, Feng Y, Xiao L	2021	Parasitology Research	Scopus
254	Molecular Detection and Characterization of Blastocystis sp. and Enterocytozoon bieneusi in Cattle in Northern Spain	Abarca N, Santin M, Ortega S, Maloney JG, George NS, Molokin A, Cardona GA, Dashti A, Koster PC, Bailo B, Hernandez-de-Mingo M, Muadica AS, Calero-Bernal R, Carmena D, Gonzalez-Barrio D	2021	Veterinary Sciences	Scopus
255	Next-generation sequencing reveals wide genetic diversity of Blastocystis subtypes in chickens including potentially zoonotic subtypes	Maloney JG, Cunha MJRD, Cunha MJRD, Cunha MJRD, Molokin A, Cury MC, Santin M	2021	Parasitology Research	PubMed
256	Subtyping of Blastocystis infection in common quails (Coturnix coturnix) in Xinxiang city of China	Liu X, Yang X, An Z, Ge Y, Wang R, Dong H, Zhu H	2021	The Thai journal of veterinary medicine	PubMed

S/N	Title	Authors	Year	Journal	Search Source
257	Worldwide prevalence of emerging parasite Blastocystis in immunocompromised patients: A systematic review and meta-analysis	Khorshidvand Z, Khazaei S, Amiri M, Taherkhani H, Mirzaei A	2021	Microbial Pathogenesis	Google Scholar
258	Molecular prevalence and subtype distribution of Blastocystis sp. in Asia and in Australia.	Nemati S, Zali MR, Johnson P, Mirjalali H, Karanis P	2021	Journal of Water and Health	Scopus
259	Genetic characterization of Blastocystis from wild animals in Sichuan Wolong National Natural Reserve, southwestern of China-Zoonotic potential	Chen S, Meng W, Zhou Z, Deng L, Shi X, Chai Y, Liu H, Cheng Y, Zhong Z, Fu H, Shen L, Zhang K, He T, Peng G	2021	The article you are looking for was published in the journal Parasites & Vector	PubMed
260	New insights into the interactions between Blastocystis, the gut microbiota, and host immunity	Deng L, Deng L, Wojciech L, Gascoigne NRJ, Peng G, Tan KSW	2021	PLOS Pathogens	Web of Science
261	Blastocystis sp. Prevalence and Subtypes Distribution amongst Syrian Refugee Communities Living in North Lebanon.	Khaled S, Khaled S, Gantois N, Ayoubi A, Even G, Sawant M, Houmayraa JE, Nabot M, Benamrouz-Vanneste S, Chabe M, Certad G, Safadi DE, Dabboussi F, Hamze M, Viscogliosi E	2021	Microorganisms	Scopus
262	Colorectal cancer and Blastocystis sp. infection.	Sulżyc-Bielicka, V., Kołodziejczyk, L., Adamska, M., Skotarczak, B., Jaczewska, S., Safranow, K., Bielicki, P., Kładny, J., & Bielicki, D	2021	Parasites & Vectors	Web of Science

S/N	Title	Authors	Year	Journal	Search Source
263	Blastocystis sp. Carriage and Irritable Bowel Syndrome: Is the Association Already Established?	Salvador F, Lobo B, Goterris L, Alonso-Cotoner C, Santos J, Sulleiro E, Bailo B, Carmena D, Sanchez-Montalva A, Bosch-Nicolau P, Espinosa-Pereiro J, Fuentes I, Molina I	2021	Biology	Web of Science
264	Molecular prevalence and subtype distribution of Blastocystis sp. in Asia and in Australia.	Nemati S, Zali MR, Johnson P, Mirjalali H, Karanis P	2021	Journal of Water and Health	Scopus
265	The Current Molecular Epidemiological Scenario of Cryptosporidium, Giardia and Blastocystis in Spain. Implication for Public Health	Koster PC, Carmena D	2020	Tropical Medicine and Infectious Disease	Google Scholar
266	Genetic Diversity of Blastocystis in Kindergarten Children in Southern Xinjiang, China	Qi M, Qi M, Wei Z, Zhang Y, Zhang Q, Li J, Zhang L, Wang R	2020	Parasites & Vectors	PubMed
267	Microbiota characterization in Blastocystis-colonized and Blastocystis-free school-age children from Colombia.	Castaneda S, Castaneda S, Munoz M, Villamizar X, Hernandez PC, Vasquez LR, Tito RY, Ramirez JD	2020	Parasites & Vectors	PubMed
268	Prevalence and Subtype Distribution of Blastocystis Infection in Patients with Diabetes Mellitus in Thailand.	Popruk N, Prasongwattana S, Mahittikorn A, Palasuwan A, Popruk S, Palasuwan D	2020	International Journal of Environmental Research and Public Health	Google Scholar
269	Molecular Detection and Subtyping of Blastocystis Detected in Wild Boars (Sus scrofa) in South Korea.	Lee H, Seo M, Oem J, Kim Y, Lee S, Kim J, Jeong H, Jheong W, Kim Y, Lee W, Kwon O, Kwak D	2020	Journal of Wildlife Diseases	Scopus

S/N	Title	Authors	Year	Journal	Search Source
270	Parasitic infections in irritable bowel syndrome patients: evidence to propose a possible link, based on a case-control study in the south of Iran	Shafiei Z, Esfandiari F, Sarkari B, Rezaei Z, Fatahi MR, Asl SMKH	2020	BMC Research Notes	Scopus
271	Prevalence and subtype distribution of Blastocystis sp. in cattle from Pahang, Malaysia.	Kamaruddin, Yusof M, Mohammad	2020	Tropical biomedicine	Web of Science
272	A Study on the Prevalence and Subtype Diversity of the Intestinal Protist Blastocystis sp. in a Gut-Healthy Human Population in the Czech Republic.	Lhotská, Z., Jirků, M., Hložková, O., Brožová, K., Jirsová, D., Stensvold, C. R., Kolisko, M., & Pomajbíková, K	2020	Frontiers in Cellular and Infection Microbiology	Scopus
273	First molecular subtyping and phylogeny of Blastocystis sp. isolated from domestic and synanthropic animals (dogs, cats and brown rats) in southern Iran	Mohammadpour I, Bozorg-Ghalati F, Gazzonis AL, Manfredi MT, Motazedian MH, Mohammadpour N	2020	Parasites & Vectors	PubMed
274	An overview of some medicinal plants and isolated active compounds with potential antiprotozoal activity	Ojuromi OT, Ashafa AOT	2020	Tropical Journal of Pharmaceutical Research	PubMed
275	The Current Molecular Epidemiological Scenario of Cryptosporidium, Giardia and Blastocystis in Spain. Implication for Public Health	Koster PC, Carmena D	2020	Tropical Medicine and Infectious Disease	Scopus
276	A Study on the Prevalence and Subtype Diversity of the Intestinal Protist Blastocystis sp. in a Gut-Healthy Human Population in the Czech Republic.	Lhotská, Z., Jirků, M., Hložková, O., Brožová, K., Jirsová, D., Stensvold, C. R., Kolisko, M., & Pomajbíková, K	2020	Frontiers in Cellular and Infection Microbiology	PubMed
277	Prevalence and subtype distribution of Blastocystis sp. in cattle from Pahang, Malaysia.	Kamaruddin, Yusof M, Mohammad	2020	Tropical biomedicine	Google Scholar

S/N	Title	Authors	Year	Journal	Search Source
278	Assessment of the subtypes and the zoonotic risk of Blastocystis sp. of experimental macaques in Yunnan province, southwestern China	Li TC, Li Z, Zhang YL, Chen WJ, Dong XL, Yang JF, Lin K, Zou Y, Jiang XC, Wang L, Luo X, Zhu XQ	2020	Parasitol Res	PubMed
279	Blastocystis incidence, spontaneous clearance, persistence and risk factors in a rural community in Thailand: a prospective cohort study	Wongthamarin K, Trairattanapa T, Kijanukul S, Kritsilpe T, Poobunjirdkul S, Chuengdee W, Taamasri P, Leelayoova S, Mungthin M	2020	Asian Pac J Trop Med	PubMed
280	Molecular epidemiology of Blastocystis in urban and periurban human populations in Arequipa, Peru	Ascuna-Durand K, Salazar-Sanchez R, Cartillo-Neyra R, Cartillo-Neyra R, Ballon-Echegaray J	2020	medRxiv	Web of Science
281	First molecular subtyping and phylogeny of Blastocystis sp. isolated from domestic and synanthropic animals (dogs, cats and brown rats) in southern Iran	Mohammadpour I, Bozorg-Ghalati F, Gazzonis AL, Manfredi MT, Motazedian MH, Mohammadpour N	2020	Parasites & Vectors	Scopus
282	Contrasting microbiota profiles observed in children carrying either Blastocystis spp. or the commensal amoebas Entamoeba coli or Endolimax nana.	Alzate JF, Toro-Londono MA, Cabarcas F, Garcia-Montoya GM, Galvan-Diaz AL	2020	Scientific Reports	Scopus
283	Genetic Diversity of Blastocystis in Kindergarten Children in Southern Xinjiang, China	Qi M, Qi M, Wei Z, Zhang Y, Zhang Q, Li J, Zhang L, Wang R	2020	Parasites & Vectors	Web of Science
284	Blastocystis spp. subtype 10 infected beef cattle in Kamal and Socah, Bangkalan, Madura, Indonesia.	Suwanti LT, Susana Y, Hastutiek P, Suprihati E, Lastuti NDR	2020	Veterinary World	Google Scholar
285	Parasitic infections in irritable bowel syndrome patients: evidence to propose a possible link, based on a case-control study in the south of Iran	Shafiei Z, Esfandiari F, Sarkari B, Rezaei Z, Fatahi MR, Asl SMKH	2020	BMC Research Notes	Scopus
286	Assessment of the subtypes and the zoonotic risk of Blastocystis sp. of experimental macaques in Yunnan province, southwestern China	Li TC, Li Z, Zhang YL, Chen WJ, Dong XL, Yang JF, Lin K, Zou Y, Jiang XC, Wang L, Luo X, Zhu XQ	2020	Parasitol Res	Scopus

S/N	Title	Authors	Year	Journal	Search Source
287	Blastocystis isolates from a dog and their owners presenting with chronic diarrhoea. Dogs as reservoirs of Blastocystis: research in Poland and worldwide	Kaczmarek, A., Rocka, A., Wesolowska, M., Golab, E., & Salamatin, R	2020	Annals of parasitology	Google Scholar
288	Epidemiology of Blastocystis infection from 1990 to 2019 in China	Ning C, Hu Z, Chen J, Ai L, Tian L	2020	Infectious Diseases of Poverty	Web of Science
289	Prevalence and Molecular Subtyping of Blastocystis from Patients with Irritable Bowel Syndrome, Inflammatory Bowel Disease and Chronic Urticaria in Iran	Shirvani G, Fasihi-Harandi M, Raiesi O, Bazargan N, Zahedi MJ, Sharifi I, Kalantari-Khandani B, Nooshadokht M, Shabandoust H, Mohammadi MA, Ebrahimipour M, Babaei Z	2020	Acta Parasitologica	Web of Science
290	The Current Molecular Epidemiological Scenario of Cryptosporidium, Giardia and Blastocystis in Spain. Implication for Public Health	Koster PC, Carmena D	2020	Tropical Medicine and Infectious Disease	Web of Science
291	Occurrence and genotypes of Cryptosporidium spp., Giardia duodenalis, and Blastocystis sp. in household, shelter, breeding, and pet market dogs in Guangzhou, southern China.	Shenquan L, Lin X, Sun Y, Nanshan Q, Lv M, Caiyan W, Juan L, Junjing H, Yu L, Haiming C, Wenwan X, Mingfei S, Li G	2020	Scientific Reports	Scopus
292	Blastocystis spp.: modern views on classification and pathogenic properties	Shahinian VR, Kharchenko NV, Danko OP, Kharchenko VV, Sopil HV	2020	Modern Gastroenterology	Web of Science
293	Nitazoxanide: Jack of All, Master of None?	Bolia R	2020	Indian Journal of Pediatrics	PubMed
294	Differentiation of Blastocystis and parasitic archamoebids encountered in untreated wastewater samples by amplicon-based next-generation sequencing	Stensvold CR, Lebbad M, Hansen A, Beser J, Belkessa S, Belkessa S, Andersen LO, Clark CG	2020	Parasite Epidemiology and Control	Scopus

S/N	Title	Authors	Year	Journal	Search Source
295	Occurrence of Blastocystis-subtypes in patients from Italy revealed association of ST3 with a healthy gut microbiota	Gabrielli S, Gabrielli S, Furzi F, Sulekova LF, Taliani G, Mattiucci S, Mattiucci S	2020	Parasite Epidemiology and Control	Web of Science
296	Blastocystis, urticaria, and skin disorders: review of the current evidences	Bahrami F, Babaei E, Badirzadeh A, Riabi TR, Abdoli A	2020	European Journal of Clinical Microbiology & Infectious Diseases	Google Scholar
297	Epidemiology of Blastocystis sp. infection in China: a systematic review	Deng L, Chai Y, Zhou Z, Liu H, Zhong Z, Hu Y, Fu H, Yue C, Peng G	2019	Parasite	Google Scholar
298	Detecção de DNA de Blastocystis sp. em pacientes candidatos a transplantes atendidos no Hospital das Clínicas da Faculdade de Medicina da Universidade de São Paulo (HCFMUSP)	Silva, M. R. A. S	2019	Universidade de São Paulo (USP)	Google Scholar
299	Giardia duodenalis in the UK: current knowledge of risk factors and public health implications.	Horton B, Bridle H, Alexander CL, Katzer F	2019	Parasitology	Scopus
300	First report of Blastocystis in giant pandas, red pandas, and various bird species in Sichuan province, southwestern China.	Deng L, Yao J, Liu H, Zhou Z, Chai Y, Wang W, Zhong Z, Deng J, Ren Z, Fu H, Yan X, Yue C, Peng G	2019	International journal for parasitology. Parasites and wildlife	Google Scholar
301	Interactions between a pathogenic Blastocystis subtype and gut microbiota: in vitro and in vivo studies	Yason JA, Yason JA, Liang YR, Png CW, Zhang Y, Tan KSW, Tan KSW	2019	Microbiome	Scopus

S/N	Title	Authors	Year	Journal	Search Source
302	Determinación in vitro de la sensibilidad a antiparasitarios de Blastocystis spp aislados de animales domésticos en la ciudad de Cúcuta [In vitro determination of the sensitivity to antiparasitics of Blastocystis spp isolated from domestic animals in the city of Cúcuta]	Mendoza JJB, Pabon JHN	2019	Universidad de Pamplona	Web of Science
303	Molecular characterization of Blastocystis sp. in captive wildlife in Bangladesh National Zoo: Non-human primates with high prevalence and zoonotic significance.	Li J, Li J, Karim MR, Li D, Sumon SMMR, Siddiki SF, Rume FI, Sun R, Jia Y, Zhang L	2019	International journal for parasitology. Parasites and wildlife	Scopus
304	Molecular epidemiology of Blastocystis isolated from animals in the state of Rio de Janeiro, Brazil.	Valença-Barbosa, C., Bomfim, T. C. B. D., Teixeira, B. R., Gentile, R., Neto, S. F. D. C., Magalhães, B. S. N., Balthazar, D. D. A., Silva, F. A. D., Biot, R., Levy, C. M. D., & Santos, H. L. C	2019	PLOS ONE	Scopus
305	Determinación in vitro de la sensibilidad a antiparasitarios de Blastocystis spp aislados de animales domésticos en la ciudad de Cúcuta [In vitro determination of the sensitivity to antiparasitics of Blastocystis spp isolated from domestic animals in the city of Cúcuta]	Mendoza JJB, Pabon JHN	2019	Universidad de Pamplona	Google Scholar
306	Evaluation of the effect of 1,3-bis-(4-phenyl-[1,2,3] triazole-1-il)2-propanol in comparison with metronidazole in an in vitro culture of Blastocystis in samples of patients with irritable bowel syndrome.	Garcia-Flores L, Santillan-Benitez JG, Cuevas-Yanez E, Caballero-Vasquez P, Zamudio-Chavez S, Morales-Avila E	2019	Journal of Parasitic Diseases	Web of Science

S/N	Title	Authors	Year	Journal	Search Source
307	Risk factors for Blastocystis infection in HIV/AIDS patients with highly active antiretroviral therapy in Southwest China	Zhang S, Kang F, Chen J, Tian L, Geng L	2019	Infectious Diseases of Poverty	PubMed
308	A Simple Genotyping Method for Rapid Differentiation of Blastocystis Subtypes and Subtype Distribution of Blastocystis spp. in Thailand	Srichaipon N, Nuchprayoon S, Charuchaibovorn S, Sukkapan P, Sanprasert V	2019	Pathogenetics	Scopus
309	Zoonotic and genetically diverse subtypes of Blastocystis in US pre-weaned dairy heifer calves	Maloney JG, Lombard JE, Urie NJ, Shivley CB, Santin M	2019	Parasitology Research	Scopus
310	DPDx - Laboratory identification of parasites of public health concern: Blastocystis	Centers for Disease Control and Prevention	2019	CDC Resource	Web of Science
311	Determination the subtypes of Blastocystis sp. and evaluate the effect of these subtypes on pathogenicity	Cakir F, Cicek M, Yildirim IH	2019	Acta Parasitol	Web of Science
312	Higher prevalence of Blastocystis hominis in healthy individuals than patients with gastrointestinal symptoms from Ahvaz, southwestern Iran	Kataki MM, Tavalla M, Beiromvand M	2019	Comparative Immunology Microbiology and Infectious Diseases	Web of Science
313	Blastocystis ST-1 is associated with irritable bowel syndrome-diarrhoea (IBS-D) in Indonesian adolescences	Kesuma Y, Firmansyah A, Bardosono S, Sari IP, Kurniawan A	2019	Parasite Epidemiol Control	Web of Science
314	Molecular characterization of Blastocystis subtypes in HIV-positive patients and evaluation of risk factors for colonization.	Sulekova LF, Gabrielli S, Furzi F, Milardi GL, Biliotti E, Angelis MD, Iaiani G, Fimiani C, Maiorano M, Mattiucci S, Taliani G	2019	BMC Infectious Diseases	Web of Science
315	Resistance towards metronidazole in Blastocystis sp.: A pathogenic consequence.	Rajamanikam A, Hooi HS, Kudva M, Samudi C, Kumar S	2019	PLOS ONE	Web of Science

S/N	Title	Authors	Year	Journal	Search Source
316	Molecular characterization of Blastocystis subtypes in HIV-positive patients and evaluation of risk factors for colonization.	Sulekova LF, Gabrielli S, Furzi F, Milardi GL, Biliotti E, Angelis MD, Iaiani G, Fimiani C, Maiorano M, Mattiucci S, Taliani G	2019	BMC Infectious Diseases	PubMed
317	A hospital qPCR-based survey of 10 gastrointestinal parasites in routine diagnostic screening, Marseille, France.	Menu E, Mary C, Toga I, Raoult D, Ranque S, Bittar F	2019	Epidemiology and Infection	Scopus
318	Next generation amplicon sequencing improves detection of Blastocystis mixed subtype infections	Maloney JG, Molokin A, Santin M	2019	Infection, Genetics and Evolution	Web of Science
319	Next generation amplicon sequencing improves detection of Blastocystis mixed subtype infections	Maloney JG, Molokin A, Santin M	2019	Infection, Genetics and Evolution	Google Scholar
320	A summary of Blastocystis subtypes in North and South America	Jimenez P, Jaimes JE, Ramirez JD	2019	Parasites & Vectors	PubMed
321	Resistance towards metronidazole in Blastocystis sp.: A pathogenic consequence.	Rajamanikam A, Hooi HS, Kudva M, Samudi C, Kumar S	2019	PLOS ONE	PubMed
322	Blastocytosis hominis: Unboxing Its Clinical Significance	Rozi MF, Darlan DM	2019	Sumatera Medical Journal	Web of Science
323	DPDx - Laboratory identification of parasites of public health concern: Blastocystis	Centers for Disease Control and Prevention	2019	CDC Resource	PubMed
324	Use of next-generation amplicon sequencing to study Blastocystis genetic diversity in a rural human population from Mexico.	Rojas-Velazquez L, Maloney JG, Molokin A, Moran P, Serrano-Vazquez A, Gonzalez E, Perez-Juarez H, Ximenez C, Santin M	2019	Parasites & Vectors	Scopus

S/N	Title	Authors	Year	Journal	Search Source
325	Blastocystis sp. puesta al dia sobre su papel parasitario	Bastidas G, Malave C, Bastidas D	2019	Gaceta Médica Boliviana	Scopus
326	Epidemiology of Blastocystis sp. infection in China: a systematic review	Deng L, Chai Y, Zhou Z, Liu H, Zhong Z, Hu Y, Fu H, Yue C, Peng G	2019	Parasite	PubMed
327	Higher prevalence of Blastocystis hominis in healthy individuals than patients with gastrointestinal symptoms from Ahvaz, southwestern Iran	Kataki MM, Tavalla M, Beiromvand M	2019	Comparative Immunology Microbiology and Infectious Diseases	Scopus
328	A hospital qPCR-based survey of 10 gastrointestinal parasites in routine diagnostic screening, Marseille, France.	Menu E, Mary C, Toga I, Raoult D, Ranque S, Bittar F	2019	Epidemiology and Infection	Web of Science
329	Risk factors for Blastocystis infection in HIV/AIDS patients with highly active antiretroviral therapy in Southwest China	Zhang S, Kang F, Chen J, Tian L, Geng L	2019	Infectious Diseases of Poverty	Scopus
330	Impact of pH on the viability and morphology of Blastocystis isolates.	Haziqah F, Chandrawathani, Douadi, Suresh, Wilson, Khalid M, Rajamanikam, Lewis, Zain M	2018	Tropical biomedicine	Scopus
331	PCR-based molecular characterization of Blastocystis hominis subtypes in southwest of Iran	Khademvatan S, Masjedizadeh R, Yousefi-Razin E, Mahbodfar H, Rahim F, Yousefi E, Foroutan M, Foroutan M	2018	Journal of Infection and Public Health	Web of Science
332	Genetic diversity and pathogenicity of Blastocystis.	Skotarczak B	2018	Annals of Agricultural and Environmental Medicine	Scopus

S/N	Title	Authors	Year	Journal	Search Source
333	Blastocystis subtype 5: Predominant subtype on pig farms, Thailand.	Pintong, Sunyanusin, Prasertbun, Mahittikorn, Mori, Changbunjong, Komalamisra, Sukthana, Popruk	2018	Parasitology international	Scopus
334	Blastocystis hominis transmission by non-potable water: a case report in Italy	Angelici MC, Nardis C, Scarpelli R, Ade P	2018	New Microbiologica	Web of Science
335	Additional Glance on the Role of Dientamoeba fragilis & Blastocystis hominis in Patients with Irritable Bowel Syndrome.	Ibrahim AN, Al-Ashkar AM, Nazeer JT	2018	Iranian Journal of Parasitology	Scopus
336	High diversity of Blastocystis subtypes isolated from asymptomatic adults living in Chiang Rai, Thailand.	Yowang A, Tsaousis AD, Chumphonsuk T, Thongsin N, Kullawong N, Popluechai S, Gentekaki E	2018	Infection, Genetics and Evolution	PubMed
337	Subtype distribution of Blastocystis isolated from humans and associated animals in an indigenous community with poor hygiene in Peninsular Malaysia	Mohammad N, Al-Mekhlafi HM, Anuar TS	2018	Tropical Biomedicine	PubMed
338	Blastocystis infection and subtype distribution in humans, cattle, goats, and pigs in central and western Thailand	Udonsom R, Prasertbun R, Mahittikorn A, Mori H, Changbunjong T, Komalamisra C, Pintong AR, Sukthana Y, Popruk S	2018	Infection, Genetics and Evolution	Web of Science
339	Asymptomatic Intestinal Colonization with Protist Blastocystis Is Strongly Associated with Distinct Microbiome Ecological Patterns	Nieves-Ramirez ME, Nieves-Ramirez ME, Partida-Rodriguez O, Partida-Rodriguez O, Laforest-Lapointe I, Reynolds LA, Reynolds LA, Brown EM, Valdez-Salazar A, Moran-Silva P, Rojas-Velazquez L, Morien E, Parfrey LW, Jin M, Jin M, Walter J, Torres J, Arrieta M, Arrieta M, Ximenez-Garcia C, Finlay BB	2018	mSystems	Scopus

S/N	Title	Authors	Year	Journal	Search Source
340	Occurrence and subtype distribution of Blastocystis sp. in humans, dogs and cats sharing household in northern Spain and assessment of zoonotic transmission risk.	Paulos S, Koster PC, Lucio AD, Hernandez-de-Mingo M, Cardona GA, Fernandez-Crespo JC, Stensvold CR, Carmena D	2018	Zoonoses and Public Health	Scopus
341	Blastocystis hominis transmission by non-potable water: a case report in Italy	Angelici MC, Nardis C, Scarpelli R, Ade P	2018	New Microbiologica	Web of Science
342	Prevalence of intestinal parasites, with emphasis on the molecular epidemiology of Giardia duodenalis and Blastocystis sp., in the Paranagua Bay, Brazil: a community survey.	Segui R, Munoz-Antoli C, Klisiowicz DDR, Oishi CY, Koster PC, Lucio AD, Hernandez-de-Mingo M, Puente P, Toledo R, Esteban JG, Carmena D	2018	Parasites & Vectors	Scopus
343	Blastocystis subtypes detected in long-tailed macaques in Thailand-Further evidence of cryptic host specificity.	Vaisusuk, Saijuntha, Sedlak, Thanchomnang, Pilap, Suksavate, Stensvold, Tantrawatpan	2018	Acta tropica	PubMed
344	Asymptomatic Intestinal Colonization with Protist Blastocystis Is Strongly Associated with Distinct Microbiome Ecological Patterns	Nieves-Ramirez ME, Nieves-Ramirez ME, Partida-Rodriguez O, Partida-Rodriguez O, Laforest-Lapointe I, Reynolds LA, Reynolds LA, Brown EM, Valdez-Salazar A, Moran-Silva P, Rojas-Velazquez L, Morien E, Parfrey LW, Jin M, Jin M, Walter J, Torres J, Arrieta M, Arrieta M, Ximenez-Garcia C, Finlay BB	2018	mSystems	Scopus
345	Molecular detection and subtyping of Blastocystis in Javan rusa (Cervus timorensis) and sika deer (Cervus nippon) from Peninsular Malaysia	Mohammad NA, Al-Mekhlafi HM, Moktar N, Anuar TS	2018	The Thai journal of veterinary medicine	Scopus

S/N	Title	Authors	Year	Journal	Search Source
346	The role of Blastocystis hominis in the activation of ulcerative colitis.	Kok M, Cekin Y, cekin AH, Uyar S, Harmandar FA, Sahinturk Y	2018	The Turkish journal of gastroenterology	Scopus
347	The role of Blastocystis hominis in the activation of ulcerative colitis.	Kok M, Cekin Y, cekin AH, Uyar S, Harmandar FA, Sahinturk Y	2018	The Turkish journal of gastroenterology	Web of Science
348	Molecular detection and subtyping of Blastocystis in Javan rusa (Cervus timorensis) and sika deer (Cervus nippon) from Peninsular Malaysia	Mohammad NA, Al-Mekhlafi HM, Moktar N, Anuar TS	2018	The Thai journal of veterinary medicine	PubMed
349	Subtype distribution of Blastocystis isolated from humans and associated animals in an indigenous community with poor hygiene in Peninsular Malaysia.	Mohammad, Al-Mekhlafi, Anuar	2018	Tropical biomedicine	Scopus
350	Additional Glance on the Role of Dientamoeba fragilis & Blastocystis hominis in Patients with Irritable Bowel Syndrome.	Ibrahim AN, Al-Ashkar AM, Nazeer JT	2018	Iranian Journal of Parasitology	Web of Science
351	Molecular detection and subtyping of Blastocystis in Javan rusa (Cervus timorensis) and sika deer (Cervus nippon) from Peninsular Malaysia	Mohammad NA, Al-Mekhlafi HM, Moktar N, Anuar TS	2018	The Thai journal of veterinary medicine	Scopus
352	Blastocystis subtypes detected in long-tailed macaques in Thailand-Further evidence of cryptic host specificity.	Vaisusuk, Saijuntha, Sedlak, Thanchomnang, Pilap, Suksavate, Stensvold, Tantrawatpan	2018	Acta tropica	Scopus
353	Blastocystis subtypes and their association with Irritable Bowel Syndrome	Cifre S, Gozalbo M, Ortiz V, Soriano JM, Merino JF, Trelis M	2018	Medical Hypotheses	PubMed
354	Blastocystis subtype 5: Predominant subtype on pig farms, Thailand.	Pintong, Sunyanusin, Prasertbun, Mahittikorn, Mori, Changbunjong, Komalamisra, Sukthana, Popruk	2018	Parasitology international	Web of Science

S/N	Title	Authors	Year	Journal	Search Source
355	Exacerbated symptoms in Blastocystis sp.-infected patients treated with metronidazole: two case studies.	Rajamanikam A, Kumar S, Samudi C, Kudva M	2018	Parasitology Research	PubMed
356	Large-scale comparative metagenomics of Blastocystis , a common member of the human gut microbiome	Beghini, F., Pasolli, E., Truong, T. D., Putignani, L., Cacciò, S. M., & Segata, N	2017	The ISME Journal	Google Scholar
357	Molecular Epidemiology of Giardia, Blastocystis and Cryptosporidium among Indigenous Children from the Colombian Amazon Basin	Cian, A., El Safadi, D., Osman, M., Moriniere, R., Gantois, N., Benamrouz-Vanneste, S., Delgado-Viscogliosi, P., Guyot, K., Li, L., Monchy, S., Noël, C., Poirier, P., Nourrisson, C., Wawrzyniak, I., Delbac, F., Bosc, S., Chabé, M., Petit, T., Certad, G., & Viscogliosi, E	2017	Frontiers in Microbiology	Google Scholar
358	Low efficacy of metronidazole in the eradication of Blastocystis hominis in symptomatic patients: Case series and systematic literature review	Batista L, Jove JP, Rosinach M, Gonzalo V, Sainz E, Loras C, Forne M, Esteve M, Fernandez-Banares F	2017	Gastroenterologia y Hepatologia	Scopus
359	Comparative Prevalence of Blastocystis in Patients with the Irritable Bowel Syndrome and Healthy Individuals: A Case Control Study	Beiromvand M, Hashemi SJ, Arjmand R, Sadjadei N, Hardanipasand L	2017	Jundishapur Journal of Microbiology	Web of Science
360	Blastocystis: how do specific diets and human gut microbiota affect its development and pathogenicity?	Lepczynska M, Bialkowska J, Dzika E, Piskorz-Ogorek K, Piskorz-Ogorek K, Korycinska J	2017	European Journal of Clinical Microbiology & Infectious Diseases	Web of Science
361	Comparative Prevalence of Blastocystis in Patients with the Irritable Bowel Syndrome and Healthy Individuals: A Case Control Study	Beiromvand M, Hashemi SJ, Arjmand R, Sadjadei N, Hardanipasand L	2017	Jundishapur Journal of Microbiology	Web of Science

S/N	Title	Authors	Year	Journal	Search Source
362	Genotyping of Enterocytozoon bieneusi and Subtyping of Blastocystis in Cancer Patients: Relationship to Diarrhea and Assessment of Zoonotic Transmission.	Zhang W, Ren G, Zhao W, Yang Z, Shen Y, Sun Y, Liu A, Cao J	2017	Frontiers in Microbiology	Scopus
363	Molecular epidemiology of blastocystosis in Malaysia: Does seasonal variation play an important role in determining the distribution and risk factors of Blastocystis subtype infections in the Aboriginal community?	Noradilah, Moktar, Anuar, Lee, Salleh, Manap, Mohtar, Azrul, Abdullah, Nordin, Abdullah	2017	Parasites & vectors	Scopus
364	Erratum to: the role of Blastocystis sp. and Dientamoeba fragilis in irritable bowel syndrome: a systematic review and meta-analysis.	Rostami A, Rostami A, Riahi SM, Riahi SM, Haghighi A, Saber V, Armon B, Seyyedtabaei SJ	2017	Parasitology Research	Scopus
365	Blastocystis in Côte d'Ivoire: Molecular identification and epidemiological data	D'Alfonso R, Santoro M, Essi D, Monsia A, Kabore Y, Gle C, Cave DD, Sorge R, Cristanziano VD, Berrilli F	2017	European Journal of Clinical Microbiology & Infectious Diseases	Web of Science
366	Comparative Prevalence of Blastocystis in Patients with the Irritable Bowel Syndrome and Healthy Individuals: A Case Control Study	Beiromvand M, Hashemi SJ, Arjmand R, Sadjadei N, Hardanipasand L	2017	Jundishapur Journal of Microbiology	Scopus
367	Targeted metagenomic sequencing data of human gut microbiota associated with Blastocystis colonization.	Siegwald L, Audebert C, Even G, Viscogliosi E, Caboche S, Chabe M	2017	Scientific Data	Scopus
368	Predominance and association risk of Blastocystis hominis subtype I in colorectal cancer: a case control study	Mohamed A, Mohamed A, Ahmed MA, Ahmed SA, Al-Semany SA, Alghamdi S, Zaglool DAM	2017	Infectious Agents and Cancer	Google Scholar

S/N	Title	Authors	Year	Journal	Search Source
369	Molecular Epidemiology of Blastocystis sp. in Various Animal Groups from Two French Zoos and Evaluation of Potential Zoonotic Risk	Cian, A., El Safadi, D., Osman, M., Moriniere, R., Gantois, N., Benamrouz-Vanneste, S., Delgado-Viscogliosi, P., Guyot, K., Li, L., Monchy, S., Noël, C., Poirier, P., Nourrisson, C., Wawrzyniak, I., Delbac, F., Bosc, S., Chabé, M., Petit, T., Certad, G., & Viscogliosi, E	2017	PLOS ONE	Web of Science
370	Prevalence and risk factors of Blastocystis infection among underprivileged communities in rural Malaysia	Mohammad NA, Al-Mekhlafi HM, Moktar N, Anuar TS	2017	Asian Pac J Trop Med	Google Scholar
371	Blastocystis in Côte d'Ivoire: Molecular identification and epidemiological data	D'Alfonso R, Santoro M, Essi D, Monsia A, Kabore Y, Gle C, Cave DD, Sorge R, Cristanziano VD, Berrilli F	2017	European Journal of Clinical Microbiology & Infectious Diseases	Scopus
372	Extreme genome diversity in the hyper-prevalent parasitic eukaryote Blastocystis	Gentekaki, E., Curtis, B. A., Stairs, C. W., Klimes, V., Elias, M., Salas-Leiva, D. E., Herman, E. K., Eme, L., Arias, M. C., Henrissat, B., Hilliou, F., Klute, M. J., Suga, H., Malik, S. B., Pightling, A. W., Kolisko, M., Rachubinski, R. A., Schlacht, A., Soanes, D. M., & Roger, A. J	2017	PLOS Biology	Google Scholar
373	Blastocystis and irritable bowel syndrome: Frequency and subtypes from Iranian patients	Khademvatan S, Khademvatan S, Masjedizadeh R, Rahim F, Mahbodfar H, Salehi R, Yousefi-Razin E, Foroutan M	2017	Parasitology International	Google Scholar

S/N	Title	Authors	Year	Journal	Search Source
374	Molecular epidemiology of blastocystosis in Malaysia: does seasonal variation play an important role in determining the distribution and risk factors of Blastocystis subtype infections in the Aboriginal community?	Noradilah SA, Noradilah SA, Moktar N, Anuar TS, Lee IL, Salleh FM, Manap SNAA, Mohtar NSHM, Azrul SM, Abdullah WO, Nordin A, Abdullah SR	2017	Parasites & Vectors	Web of Science
375	Molecular epidemiology of Blastocystis SP in animals reared by the aborigines during wet and dry seasons in rural communities, pahang, Malaysia	Noradilah SA, Anuar TS, Moktar N, Lee IL, Salleh FM, Azreen SN, Husnie NSMM, Azrul SM, Abdullah WO, Nordin A, Abdullah SR	2017	Southeast Asian Journal of Tropical Medicine and Public Health	Scopus
376	Epidemiological and clinical profile of adult patients with Blastocystis sp. infection in Barcelona, Spain	Salvador F, Sulleiro E, Sanchez-Montalva A, Alonso C, Alonso C, Santos J, Santos J, Fuentes I, Molina I	2016	Parasites & Vectors	Scopus
377	On Blastocystis secreted cysteine proteases: a legumain-activated cathepsin B increases paracellular permeability of intestinal Caco-2 cell monolayers	Nourrisson C, Wawrzyniak I, Cian A, Livrelli V, Viscogliosi E, Delbac F, Poirier P	2016	Parasitology	Web of Science
378	Subtype Distribution of Blastocystis in Communities along the Chao Phraya River, Thailand.	Palasuwan A, Palasuwan D, Mahittikorn A, Chiabchalard R, Combes V, Popruk S	2016	Korean Journal of Parasitology	Scopus
379	Association between Blastocystis hominis and irritable bowel syndrome(IBS)	Darabian A, Berenji F, Ganji A, Fata A, Jarahi L	2016	International Journal of Medical Research and Health Sciences	Web of Science
380	Molecular epidemiology and genetic diversity of Blastocystis infection in humans in Italy	Mattiucci S, Crisafi B, Gabrielli S, Paoletti M, Cancrini G	2016	Epidemiology and Infection	Google Scholar

S/N	Title	Authors	Year	Journal	Search Source
381	Subtype analysis of Blastocystis sp. isolates from human and canine hosts in an urban community in the Philippines	Belleza ML, Reyes JC, Tongol-Rivera PN, Rivera WL	2016	Parasitol Int	Google Scholar
382	Molecular Identification and Subtype Analysis of Blastocystis	Stensvold CR, Clark CG	2016	Current protocols in microbiology	Web of Science
383	Increase number of mitochondrion-like organelle in symptomatic Blastocystis subtype 3 due to metronidazole treatment	Raman K, Kumar S, Chye TT	2016	Parasitology Research	PubMed
384	Comparison of faecal microbiota in Blastocystis-positive and Blastocystis-negative irritable bowel syndrome patients	Nagel R, Traub RJ, Allcock RJ, Kwan M, Bielefeldt-Ohmann H	2016	Microbiome	PubMed
385	Occurrence of Blastocystis sp. in water catchments at Malay villages and Aboriginal settlement during wet and dry seasons in Peninsular Malaysia	Noradilah, Lee, Anuar, Salleh, Manap A, Mohtar M, Azrul, Abdullah, Moktar	2016	PeerJ	Web of Science
386	The role of Blastocystis sp. and Dientamoeba fragilis in irritable bowel syndrome: a systematic review and meta-analysis	Rostami A, Riahi S, Haghighi A, Saber V	2016	Parasitol Res	Google Scholar
387	Increase number of mitochondrion-like organelle in symptomatic Blastocystis subtype 3 due to metronidazole treatment	Raman K, Kumar S, Chye TT	2016	Parasitology Research	Scopus
388	On Blastocystis secreted cysteine proteases: a legumain-activated cathepsin B increases paracellular permeability of intestinal Caco-2 cell monolayers	Nourrisson C, Wawrzyniak I, Cian A, Livrelli V, Viscogliosi E, Delbac F, Poirier P	2016	Parasitology	PubMed
389	Subtype Distribution of Blastocystis in Communities along the Chao Phraya River, Thailand.	Palasuwan, Palasuwan, Mahittikorn, Chiabchalard, Combes, Popruk	2016	The Korean journal of parasitology	Scopus

S/N	Title	Authors	Year	Journal	Search Source
390	Molecular Identification and Subtype Analysis of Blastocystis	Stensvold CR, Clark CG	2016	Current protocols in microbiology	PubMed
391	Association between Blastocystis hominis and irritable bowel syndrome(IBS)	Darabian A, Berenji F, Ganji A, Fata A, Jarahi L	2016	International Journal of Medical Research and Health Sciences	Web of Science
392	Immunopathological assessments of human Blastocystis spp. in experimentally infected immunocompetent and immunosuppresed mice.	Abdel-Hafeez EH, Ahmad AK, Abdelgelil NH, Abdellatif MZM, Kamal AM, Hassanin KM, Abdel-Razik AH, Abdel-Raheem EM	2016	Parasitology Research	PubMed
393	Contribution of Blastocystishominis subtypes and associated inflammatory factors in development of irritable bowel syndrome	Azizian M, Basati G, Abangah G, Mahmoudi MR, Mirzaei A	2016	Parasitology Research	Web of Science
394	Occurrence of Blastocystis sp. in water catchments at Malay villages and Aboriginal settlement during wet and dry seasons in Peninsular Malaysia	Noradilah, Lee, Anuar, Salleh, Manap A, Mohtar M, Azrul, Abdullah, Moktar	2016	PeerJ	Web of Science
395	Subtype analysis of Blastocystis sp. isolates from asymptomatic individuals in an urban community in the Philippines	Adao DEV, Serna AOD, Belleza MLB, Bolo NR, Rivera WL	2016	Annals of parasitology	Scopus
396	Molecular survey of Blastocystis sp. from humans and associated animals in an Indonesian community with poor hygiene	Yoshikawa H, Tokoro M, Nagamoto T, Arayama S, Asih PB, Rozi IE, Syafruddin D	2016	Parasitol Int	Web of Science

S/N	Title	Authors	Year	Journal	Search Source
397	[Frequency and in vitro susceptibility antiparasitic of Blastocystis hominis from patients admitted to the Hospital Regional Lambayeque, Peru].	Silva-Diaz H, Flores-Esqueche L, Llatas-Cancino D, G GV, Silva-Garcia T	2016	Revista de gastroenterologia del Peru : organo oficial de la Sociedad de Gastroenterologia del Peru	PubMed
398	Comparison of faecal microbiota in Blastocystis-positive and Blastocystis-negative irritable bowel syndrome patients	Nagel R, Traub RJ, Allcock RJ, Kwan M, Bielefeldt-Ohmann H	2016	Microbiome	Web of Science
399	Molecular epidemiology and genetic diversity of Blastocystis infection in humans in Italy	Mattiucci S, Crisafi B, Gabrielli S, Paoletti M, Cancrini G	2016	Epidemiology and Infection	PubMed
400	Molecular Identification and Subtype Analysis of Blastocystis.	Stensvold CR, Clark CG	2016	Current protocols in microbiology	Scopus
401	The role of Blastocystis sp. and Dientamoeba fragilis in irritable bowel syndrome: a systematic review and meta-analysis	Rostami A, Riahi S, Haghighi A, Saber V	2016	Parasitol Res	PubMed
402	Epidemiological and clinical profile of adult patients with Blastocystis sp. infection in Barcelona, Spain	Salvador F, Sulleiro E, Sanchez-Montalva A, Alonso C, Alonso C, Santos J, Santos J, Fuentes I, Molina I	2016	Parasites & Vectors	PubMed
403	Mysterious chronic urticaria caused by Blastocystis spp.	Lepczynska M, Chen W, Chen W, Dzika E	2016	International Journal of Dermatology	Web of Science

S/N	Title	Authors	Year	Journal	Search Source
404	Prevalence and genotype analysis of blastocystis hominis in Iran: a systematic review and meta-analysis	Badparva E, Ezatpour B, Mahmoudvand H, Behzadifar M, Behzadifar M, Kheirandish F	2016	Archives of Clinical Infectious Diseases	Scopus
405	Molecular characterization and subtyping of Blastocystis species in Irritable Bowel Syndrome patients from north India.	Das R, Khalil S, Mirdha BR, Makharia GK, Dattagupta S, Chaudhry R	2016	PLOS ONE	Google Scholar
406	Prevalence, risk factors for infection and subtype distribution of the intestinal parasite Blastocystis sp. from a large-scale multi-center study in France	Safadi DE, Cian A, Nourrisson C, Pereira B, Morelle C, Bastien P, Bellanger A, Botterel F, Candolfi E, Desoubeaux G, Lachaud L, Morio F, Pomares C, Rabodonirina M, Wawrzyniak I, Delbac F, Gantois N, Certad G, Delhaes L, Poirier P, Viscogliosi E	2016	BMC Infectious Diseases	PubMed
407	Subtype analysis of Blastocystis sp. isolates from human and canine hosts in an urban community in the Philippines	Belleza ML, Reyes JC, Tongol-Rivera PN, Rivera WL	2016	Parasitol Int	Web of Science
408	Blastocystis sp. in Irritable Bowel Syndrome (IBS) - Detection in Stool Aspirates during Colonoscopy	Ragavan ND, Kumar S, Chye TT, Mahadeva S, Shiaw-Hooi H	2015	PLOS ONE	PubMed
409	Prevalence and diagnostic approach for a neglected protozoon Blastocystis hominis	El-Marhoumy SM, EL-Nouby KA, Shoheib ZS, Salama AM	2015	Asian Pacific Journal of Tropical Disease	Scopus
410	In Vitro Antimicrobial Susceptibility Patterns of Blastocystis.	Roberts T, Roberts T, Bush SF, Ellis J, Harkness J, Stark D	2015	Antimicrobial Agents and Chemotherapy	Web of Science

S/N	Title	Authors	Year	Journal	Search Source
411	The relationship between blastocystis hominis infection and Irritable Bowel Syndrome (IBS) and comparing direct wet mount, stool culture, Formalin- Ether and trichrome staining procedures for identifying organisms	Mohemmi N, Moradi M, Khalilian A, Maghsood AH, Fallah M	2015	Bimonthly Journal of Hormozgan University of Medical Sciences	PubMed
412	Low prevalence of Blastocystis sp. in active ulcerative colitis patients	Rossen NG, Bart A, Verhaar N, Nood EV, Kootte RS, Groot PFD, D'Haens GR, Ponsioen CY, Gool TV	2015	European Journal of Clinical Microbiology & Infectious Diseases	Google Scholar
413	Are we neglecting blastocystis hominis in patients having irritable bowel syndrome	Mehta RS, Koticha A, Kuyare S, Mehta PR	2015	Journal of Evolution of medical and Dental Sciences	PubMed
414	The Interplay of Host Microbiota and Parasitic Protozoans at Mucosal Interfaces: Implications for the Outcomes of Infections and Diseases.	Bär, A., Phukan, N., Pinheiro, J., & Simoes-Barbosa, A	2015	PLOS Neglected Tropical Diseases	Web of Science
415	The role of artemether as a possible drug for treatment Blastocystis hominis infection: In vivo and in vitro studies	Zeinab HF, Eman A, Olfat H	2015	African Journal of Pharmacy and Pharmacology	Google Scholar
416	Laboratory diagnosis of Blastocystis spp. in diarrheic patients	El-Ghareeb AS, Younis MS, Fakahany AFE, Nagaty IM, Nagib MM	2015	Tropical parasitology	PubMed

S/N	Title	Authors	Year	Journal	Search Source
417	Prevalence and risk factors for Blastocystis infection among children and caregivers in a child care center, Bangkok, Thailand	Pipatsatitpong D, Leelayoova S, Mungthin M, Aunpad R, Naaglor T, Rangsin R	2015	American Journal of Tropical Medicine and Hygiene	Web of Science
418	Prevalence and risk factors for Blastocystis infection among children and caregivers in a child care center, Bangkok, Thailand	Pipatsatitpong D, Leelayoova S, Mungthin M, Aunpad R, Naaglor T, Rangsin R	2015	American Journal of Tropical Medicine and Hygiene	PubMed
419	PATHOGENICITY OF Blastocystis sp. TO THE GASTROINTESTINAL TRACT OF MICE: RELATIONSHIP BETWEEN INOCULUM SIZE AND PERIOD OF INFECTION	Pavanelli MF, Kaneshima EN, Uda CF, Colli CM, Falavigna-Guilherm AL, Gomes ML	2015	Revista Do Instituto De Medicina Tropical De Sao Paulo	Scopus
420	Drug Development Against the Major Diarrhea-Causing Parasites of the Small Intestine, Cryptosporidium and Giardia	Miyamoto Y, Eckmann L	2015	Frontiers in Microbiology	Scopus
421	In Vitro Antimicrobial Susceptibility Patterns of Blastocystis.	Roberts T, Roberts T, Bush SF, Ellis J, Harkness J, Stark D	2015	Antimicrobial Agents and Chemotherapy	Web of Science
422	Epidemiologic Study of Blastocystis Infection in an Urban Community in the Philippines	Belleza MLB, Cadacio JLC, Borja MP, Solon JAA, Padilla MA, Tongol-Rivera P, Rivera WL	2015	Journal of Environmental and Public Health	PubMed

S/N	Title	Authors	Year	Journal	Search Source
423	A novel ELISA test for laboratory diagnosis of Blastocystis spp. in human stool specimens	Dogruman-Al F, Turk S, Adiyaman-Korkmaz G, Hananel A, Levi L, Kopelowitz J, Babai O, Gross S, Greenberg Z, Herschkovitz Y, Mumcuoglu I	2015	Parasitology Research	PubMed
424	The distribution of Blastocystis subtypes in isolates from Qatar.	Abu-Madi M, Aly M, Aly M, Behnke JM, Clark CG, Balkhy HH, Balkhy HH	2015	Parasites & Vectors	PubMed
425	Blastocystis and urticaria: Examination of subtypes and morphotypes in an unusual clinical manifestation	Casero RD, Mongi F, Sanchez A, Ramirez JD	2015	Acta Tropica	Scopus
426	Molecular epidemiology of Blastocystis	Eroglu F	2015	Dicle Medical Journal	PubMed
427	Clinical significance and prevalence of Blastocystis hominis in Van, Turkey	Beyhan YE, Yilmaz H, Cengiz ZT, Ekici A	2015	Saudi Medical Journal	Google Scholar
428	Genetic Diversity of Blastocystis Isolated From Cattle in Khorramabad, Iran	Badparva E, Sadraee J, Kheirandish F	2015	Jundishapur Journal of Microbiology	PubMed
429	Prevalence and risk factors for Blastocystis infection among children and caregivers in a child care center, Bangkok, Thailand	Pipatsatitpong D, Leelayoova S, Mungthin M, Aunpad R, Naaglor T, Rangsin R	2015	American Journal of Tropical Medicine and Hygiene	Google Scholar
430	In vitro effect of some egyptian herbal extracts against blastocystis hominis	Abdel-Hafeez EH, Ahmad AK, Andelgelil NH, Abdellatif MZM, Kamal AM, Mohamed RM	2015	Journal of the Egyptian Society of Parasitology	Web of Science

S/N	Title	Authors	Year	Journal	Search Source
431	Subtype distribution of Blastocystis in Thai-Myanmar border, Thailand.	Popruk S, Udonsom R, Koompapong K, Mahittikorn A, Kusolsuk T, Ruangsittichai J, Palasuwan A	2015	Korean Journal of Parasitology	Scopus
432	Blastocystis and urticaria: Examination of subtypes and morphotypes in an unusual clinical manifestation	Casero RD, Mongi F, Sanchez A, Ramirez JD	2015	Acta Tropica	PubMed
433	Update on the pathogenic potential and treatment options for Blastocystis sp	Roberts T, Roberts T, Stark D, Harkness J, Ellis J	2014	Gut Pathogens	Web of Science
434	Molecular epidemiology of Blastocystis in pigs and their in-contact humans in Southeast Queensland, Australia, and Cambodia.	Wang, Owen, Traub, Cuttell, Inpankaew, Bielefeldt-Ohmann	2014	Veterinary parasitology	Google Scholar
435	Update on the Molecular Epidemiology and Diagnostic Tools for Blastocystis sp	Roberts T, Stark D, Harkness J, Ellis J	2014	Journal of Medical Microbiology and Diagnosis	PubMed
436	Phenotypic variation in Blastocystis sp. ST3.	Ragavan ND, Govind SK, Chye TT, Mahadeva S	2014	Parasites & Vectors	Web of Science
437	Phenotypic variation in Blastocystis sp. ST3.	Ragavan ND, Govind SK, Chye TT, Mahadeva S	2014	Parasites & Vectors	PubMed
438	Phenotypic variation in Blastocystis sp. ST3.	Ragavan ND, Govind SK, Chye TT, Mahadeva S	2014	Parasites & Vectors	Google Scholar
439	Molecular subtyping of Blastocystis spp. using a new rDNA marker from the mitochondria-like organelle genome.	Poirier P, Meloni D, Nourrisson C, Wawrzyniak I, Viscogliosi E, Livrelli V, Delbac F	2014	Parasitology	PubMed
440	Clinical pilot study: efficacy of triple antibiotic therapy in Blastocystis positive irritable bowel syndrome patients	Nagel R, Bielefeldt-Ohmann H, Traub RJ	2014	Gut Pathogens	PubMed

S/N	Title	Authors	Year	Journal	Search Source
441	Treatment failure in patients with chronic Blastocystis infection.	Roberts T, Roberts T, Ellis J, Harkness J, Marriott D, Stark D	2014	Journal of Medical Microbiology	PubMed
442	Efficacy of 5-Nitroimidazoles for the Treatment of Giardiasis: A Systematic Review of Randomized Controlled Trials	Pasupuleti V, Escobedo AA, Deshpande A, Thota P, Roman YM, Hernandez AV	2014	PLOS Neglected Tropical Diseases	Scopus
443	The pathogenic role of Blastocystis isolated from patients with irritable bowel syndrome and colitis from Iasi, Romania.	Matiut DS, Hritcu L	2014	Acta Parasitologica	Scopus
444	Molecular epidemiology of Blastocystis in pigs and their in-contact humans in Southeast Queensland, Australia, and Cambodia.	Wang, Owen, Traub, Cuttell, Inpankaew, Bielefeldt-Ohmann	2014	Veterinary parasitology	Scopus
445	Strain-Dependent Induction of Human Enterocyte Apoptosis by Blastocystis Disrupts Epithelial Barrier and ZO-1 Organization in a Caspase 3- and 9-Dependent Manner	Wu Z, Mirza H, Teo JDW, Tan KSW	2014	BioMed Research International	Scopus
446	Update on the pathogenic potential and treatment options for Blastocystis sp	Roberts T, Roberts T, Stark D, Harkness J, Ellis J	2014	Gut Pathogens	Scopus
447	Suitability of internal transcribed spacers (ITS) as markers for the population genetic structure of Blastocystis spp	Villalobos G, Orozco-Mosqueda GE, Lopez-Perez M, Lopez-Escamilla E, Cordoba-Aguilar A, Rangel-Gamboa L, Olivo-Diaz A, Romero-Valdovinos M, Maravilla P, Martinez-Hernandez F	2014	Parasites & Vectors	Scopus
448	Efficacy of 5-Nitroimidazoles for the Treatment of Giardiasis: A Systematic Review of Randomized Controlled Trials	Pasupuleti V, Escobedo AA, Deshpande A, Thota P, Roman YM, Hernandez AV	2014	PLOS Neglected Tropical Diseases	PubMed

S/N	Title	Authors	Year	Journal	Search Source
449	Patogenicidad de Blastocystis sp. Evidencias y mecanismos	Galindo LF, Gonzalez AF, Sutil YM, Perdomo YM	2014	Revista Cubana de Medicina Tropical	PubMed
450	Clinical pilot study: efficacy of triple antibiotic therapy in Blastocystis positive irritable bowel syndrome patients	Nagel R, Bielefeldt-Ohmann H, Traub RJ	2014	Gut Pathogens	Google Scholar
451	Epidemiological and diagnostic features of blastocystis infection in symptomatic patients in izmir province, Turkey.	Dağcı, H., Kurt, Ö., Demirel, M., Mandiracioğlu, A., Aydemir, Ş., Saz, U. E., Bart, A., & van Gool, T	2014	Iranian Journal of Parasitology	Scopus
452	Molecular epidemiology of Blastocystis in pigs and their in-contact humans in Southeast Queensland, Australia, and Cambodia	Wang W, Owen H, Traub RJ, Cuttell L, Inpankaew T, Bielefeldt-Ohmann H	2014	Veterinary Parasitology	Scopus
453	Suitability of internal transcribed spacers (ITS) as markers for the population genetic structure of Blastocystis spp	Villalobos G, Orozco-Mosqueda GE, Lopez-Perez M, Lopez-Escamilla E, Cordoba-Aguilar A, Rangel-Gamboa L, Olivo-Diaz A, Romero-Valdovinos M, Maravilla P, Martinez-Hernandez F	2014	Parasites & Vectors	PubMed
454	Update on the Molecular Epidemiology and Diagnostic Tools for Blastocystis sp	Roberts T, Stark D, Harkness J, Ellis J	2014	Journal of Medical Microbiology and Diagnosis	Web of Science
455	Intra-subtype variation in enteroadhesion accounts for differences in epithelial barrier disruption and is associated with metronidazole resistance in Blastocystis subtype-7.	Wu Z, Mirza H, Tan KSW	2014	PLOS Neglected Tropical Diseases	Google Scholar

S/N	Title	Authors	Year	Journal	Search Source
456	Universal health coverage: yes, but coverage of what?: the need for people-centered care	Decat P, Gyselinck K, Leyns C, Montenegro H, Criel B	2013	Tropical Medicine & International Health	Web of Science
457	Subtype identification of Blastocystis spp. isolated from patients in a major hospital in northeastern Thailand	Jantermtor S, Pinlaor P, Sawadpanich K, Pinlaor S, Sangka A, Wilailuckana C, Wongsena W, Yoshikawa H	2013	Parasitology Research	Web of Science
458	Subtype distribution of Blastocystis isolates identified in a Sydney population and pathogenic potential of Blastocystis	Roberts T, Roberts T, Stark D, Harkness J, Ellis J	2013	European Journal of Clinical Microbiology & Infectious Diseases	Web of Science
459	Recent developments in Blastocystis research.	Clark CG, Giezen MVD, Alfellani MA, Alfellani MA, Stensvold CR	2013	Advances in Parasitology	Scopus
460	Blastocystis infection in Malaysia: Evidence of waterborne and human-to-human transmissions among the Proto-Malay, Negrito and Senoi tribes of Orang Asli	Anuar TS, Ghani MKA, Azreen SN, Salleh FM, Moktar N	2013	Parasites & Vectors	Scopus
461	Amoebic forms of Blastocystis spp. - evidence for a pathogenic role	Rajamanikam A, Govind SK	2013	Parasites & Vectors	Scopus
462	Prevalence, predictors and clinical significance of Blastocystis sp. in Sebha, Libya.	Abdulsalam AM, Ithoi I, Al-Mekhlafi HM, Al-Mekhlafi HM, Khan AH, Ahmed A, Surin J, Mak JW	2013	Parasites & Vectors	Scopus
463	Prevalence, predictors and clinical significance of Blastocystis sp. in Sebha, Libya.	Abdulsalam AM, Ithoi I, Al-Mekhlafi HM, Al-Mekhlafi HM, Khan AH, Ahmed A, Surin J, Mak JW	2013	Parasites & Vectors	PubMed

S/N	Title	Authors	Year	Journal	Search Source
464	Romancing Blastocystis: A 20-Year Affair	Kumar S, Tan T	2013	Tropical Parasitology	Web of Science
465	Comparison of Sequencing (Barcode Region) and Sequence-Tagged-Site PCR for Blastocystis Subtyping	Stensvold CR	2013	Journal of Clinical Microbiology	Google Scholar
466	Identification of Blastocystis Subtype 1 Variants in the Home for Girls, Bangkok, Thailand	Thathaisong U, Siripattanapipong S, Mungthin M, Pipatsatitpong D, Tan-ariya P, Naaglor T, Leelayoova S	2013	American Journal of Tropical Medicine and Hygiene	Scopus
467	Genetic diversity of caprine Blastocystis from Peninsular Malaysia	Tan TC, Tan PC, Sharma RSK, Sugnaseelan S, Suresh K	2013	Parasitology Research	PubMed
468	Diversity and distribution of Blastocystis sp. subtypes in non-human primates	Alfellani MA, Jacob AS, Perea NO, Krecek RC, Taner-Mulla D, Verweij JJ, Levecke B, Tannich E, Clark CG, Stensvold CR	2013	Parasitology	Scopus
469	Diversity of Blastocystis subtypes in dogs in different geographical settings	Wang W, Cuttell L, Bielefeldt-Ohmann H, Inpankaew T, Inpankaew T, Owen H, Traub RJ	2013	Parasites & Vectors	Web of Science
470	Blastocystis: Genetic diversity and molecular methods for diagnosis and epidemiology	Stensvold CR	2013	Tropical parasitology	PubMed
471	Genetic diversity of blastocystis in livestock and zoo animals	Alfellani MA, Taner-Mulla D, Jacob AS, Imeede CA, Yoshikawa H, Stensvold CR, Clark CG	2013	Protist	PubMed
472	Recent developments in Blastocystis research.	Clark CG, Giezen MVD, Alfellani MA, Alfellani MA, Stensvold CR	2013	Advances in Parasitology	PubMed

S/N	Title	Authors	Year	Journal	Search Source
473	Blastocystis: Consensus of treatment and controversies	Sekar U, Shanthi M	2013	Tropical parasitology	Scopus
474	Blastocystis, an unrecognized parasite: an overview of pathogenesis and diagnosis	Wawrzyniak I, Poirier P, Viscogliosi E, Dionigia M, Texier C, Delbac F, and El Alaoui H	2013	Therapeutic Advances in Infectious Disease	Scopus
475	Infections of Blastocystis hominis and microsporidia in cancer patients: are they opportunistic?	Chandramathi S, Suresh K, Anita ZB, Kuppusamy UR	2012	Transactions of The Royal Society of Tropical Medicine and Hygiene	PubMed
476	Subtype analysis of Blastocystis isolates in Swedish patients	Forsell, J., Granlund, M., Stensvold, C. R., Clark, C. G., & Evengard, B	2012	European Journal of Clinical Microbiology & Infectious Diseases	Scopus
477	Levels of genetic diversity vary dramatically between Blastocystis subtypes.	Stensvold CR, Alfellani MA, Clark CG	2012	Infection, Genetics and Evolution	Google Scholar

S/N	Title	Authors	Year	Journal	Search Source
478	Infections of Blastocystis hominis and microsporidia in cancer patients: are they opportunistic?	Chandramathi S, Suresh K, Anita ZB, Kuppusamy UR	2012	Transactions of The Royal Society of Tropical Medicine and Hygiene	Web of Science
479	Blastocystis sp.: waterborne zoonotic organism, a possibility?	Lee LI, Chye TT, Karmacharya BM, Govind SK	2012	Parasites & Vectors	Scopus
480	A Hospital-Based Study of Epidemiological and Clinical Data on Blastocystis hominis Infection	Laodim P, Intapan PM, Sawanyawisuth K, Laummaunwai P, Maleewong W	2012	Foodborne Pathogens and Disease	Scopus
481	Blastocystis sp.: waterborne zoonotic organism, a possibility?	Lee LI, Chye TT, Karmacharya BM, Govind SK	2012	Parasites & Vectors	Web of Science
482	New Insights into Blastocystis spp.: A Potential Link with Irritable Bowel Syndrome	Poirier, P., Wawrzyniak, I., Vivarès, C. P., Delbac, F., & El Alaoui, H	2012	PLOS Pathogens	Web of Science
483	Blastocystis sp.: waterborne zoonotic organism, a possibility?	Lee LI, Chye TT, Karmacharya BM, Govind SK	2012	Parasites & Vectors	Scopus
484	Clinical Aspects of Blastocystis Infections: Advancements Amidst Controversies	Mirza H, Tan KSW	2012	Digestive Diseases and Sciences	PubMed
485	Predominance of Blastocystis sp. subtype 4 in rural communities, Nepal	Lee IL, Tan TC, Tan PC, Nanthiney DR, Biraj MK, Surendra KM, Suresh K	2012	Parasitology Research	PubMed

S/N	Title	Authors	Year	Journal	Search Source
486	Infections of Blastocystis hominis and microsporidia in cancer patients: are they opportunistic?	Chandramathi S, Suresh K, Anita ZB, Kuppusamy UR	2012	Transactions of The Royal Society of Tropical Medicine and Hygiene	Google Scholar
487	Clinical Aspects of Blastocystis Infections: Advancements Amidst Controversies	Mirza H, Tan KSW	2012	Digestive Diseases and Sciences	Web of Science
488	Blastocystis infection is associated with irritable bowel syndrome in a Mexican patient population	Jimenez-Gonzalez DE, Martinez-Flores WA, Reyes-Gordillo J, Ramirez-Miranda ME, Arroyo-Escalante S, Romero-Valdovinos M, Stark D, Souza-Saldivar V, Martinez-Hernandez F, Flisser A, Olivo-Diaz A, Maravilla P	2012	Parasitology Research	Web of Science
489	Development of metronidazole-resistant lines of Blastocystis sp.	Dunn LA, Tan KSW, Vanelle P, Juspin T, Crozet MD, Terme T, Upcroft P, Upcroft J	2012	Parasitology Research	Google Scholar
490	Blastocystis: to treat or not to treat...but how?	Engsbro AL, Stensvold CR	2012	Clinical Infectious Diseases	Google Scholar

S/N	Title	Authors	Year	Journal	Search Source
491	Subtype analysis of Blastocystis isolates in Swedish patients	Forsell, J., Granlund, M., Stensvold, C. R., Clark, C. G., & Evengard, B	2012	European Journal of Clinical Microbiology & Infectious Diseases	Scopus
492	Incidence and risk factors of Blastocystis infection in an orphanage in Bangkok, Thailand	Pipatsatitpong D, Rangsin R, Leelayoova S, Naaglor T, Mungthin M	2012	Parasites & Vectors	Scopus
493	Blastocystis infection is associated with irritable bowel syndrome in a Mexican patient population	Jimenez-Gonzalez DE, Martinez-Flores WA, Reyes-Gordillo J, Ramirez-Miranda ME, Arroyo-Escalante S, Romero-Valdovinos M, Stark D, Souza-Saldivar V, Martinez-Hernandez F, Flisser A, Olivo-Diaz A, Maravilla P	2012	Parasitology Research	Web of Science
494	Characterization of two cysteine proteases secreted by Blastocystis ST7, a human intestinal parasite	Wawrzyniak I, Texier C, Texier C, Poirier P, Poirier P, Viscogliosi E, Tan KSW, Delbac F, Delbac F, Alaoui HE, Alaoui HE	2012	Parasitology International	Web of Science
495	New Insights into Blastocystis spp.: A Potential Link with Irritable Bowel Syndrome	Poirier, P., Wawrzyniak, I., Vivarès, C. P., Delbac, F., & El Alaoui, H	2012	PLOS Pathogens	Scopus
496	Blastocystis: To treat or not to treat	Coyle CM, Varughese J, Weiss LM, Tanowitz HB	2012	Clinical Infectious Diseases	Google Scholar
497	A Hospital-Based Study of Epidemiological and Clinical Data on Blastocystis hominis Infection	Laodim P, Intapan PM, Sawanyawisuth K, Laummaunwai P, Maleewong W	2012	Foodborne Pathogens and Disease	Scopus

S/N	Title	Authors	Year	Journal	Search Source
498	A Rapid, High-Throughput Viability Assay for Blastocystis spp. Reveals Metronidazole Resistance and Extensive Subtype-Dependent Variations in Drug Susceptibilities	Mirza H, Teo JDW, Upcroft J, Tan KSW	2011	Antimicrobial Agents and Chemotherapy	Web of Science
499	Association of Blastocystis hominis genetic subtypes with urticaria	Hameed DMA, Hassanin OM, Zuel-Fakkar NM	2011	Parasitology Research	Google Scholar
500	Comparison of microscopy, culture, and conventional polymerase chain reaction for detection of Blastocystis sp. in clinical stool samples	Roberts T, Barratt J, Harkness J, Ellis J, Stark D	2011	American Journal of Tropical Medicine and Hygiene	Scopus
501	The pathogenic role of different Blastocystis hominis genotypes isolated from patients with irritable bowel syndrome	Fouad SA, Basyoni MM, Fahmy R, Kobaisi MH	2011	Arab Journal of Gastroenterology	Web of Science
502	Blastocystis sp. subtype 4 is common in Danish Blastocystis-positive patients presenting with acute diarrhea	Stensvold CR, Christiansen DB, Olsen KEP, Nielsen HV	2011	American Journal of Tropical Medicine and Hygiene	Scopus
503	Molecular subtyping of Blastocystis sp. isolates from symptomatic patients in Italy	Meloni D, Meloni D, Sanciu G, Sanciu G, Poirier P, Alaoui HE, Chabe M, Delhaes L, Dei-Cas E, Delbac F, Fiori PL, Cave DD, Viscogliosi E	2011	Parasitology Research	Scopus
504	Current Views on the Clinical Relevance of Blastocystis spp.	Tan KSW, Mirza H, Teo JDW, Wu B, MacAry PA	2010	Current Infectious Disease Reports	PubMed

S/N	Title	Authors	Year	Journal	Search Source
505	Molecular characterization of Blastocystis isolates from zoo animals and their animal-keepers	Parkar U, Traub RJ, Vitali S, Elliot A, Levecke B, Robertson ID, Geurden T, Steele J, Drake B, Thompson RA	2010	Veterinary Parasitology	PubMed
506	Short Course Combination Therapy for Giardiasis after Nitroimidazole Failure	Lopez-Velez R, Batlle C, Jimenez C, Navarro M, Norman FF, Perez-Molina JA	2010	American Journal of Tropical Medicine and Hygiene	Google Scholar
507	Differences in Clinical Significance and Morphologic Features of Blastocystis sp Subtype 3	Vassalos CM, Spanakos G, Vassalou E, Papadopoulou C, Vakalis N	2010	American Journal of Clinical Pathology	Web of Science
508	Eradication of Blastocystis carriage with antimicrobials: reality or delusion?	Stensvold CR, Smith HV, Nagel R, Olsen KEP, Traub RJ	2010	Journal of Clinical Gastroenterology	Web of Science
509	Assessment of the association between Blastocystis infection and irritable bowel syndrome.	Surangsrirat S, Thamrongwittawatpong L, Piyaniran W, Naaglor T, Khoprasert C, Taamasri P, Mungthin M, Leelayoova S	2010	Journal of the Medical Association of Thailand Chotmaihet thangphaet	PubMed
510	Differences in Clinical Significance and Morphologic Features of Blastocystis sp Subtype 3	Vassalos CM, Spanakos G, Vassalou E, Papadopoulou C, Vakalis N	2010	American Journal of Clinical Pathology	Scopus

S/N	Title	Authors	Year	Journal	Search Source
511	Assessment of the association between Blastocystis infection and irritable bowel syndrome.	Surangsrirat S, Thamrongwittawatpong L, Piyaniran W, Naaglor T, Khoprasert C, Taamasri P, Mungthin M, Leelayoova S	2010	Journal of the Medical Association of Thailand Chotmaihet thangphaet	PubMed
512	Genotypic Characterization of Blastocystis Isolates in the Philippines	Tan MAV, Rivera WL	2009	The Philippine Journal of Veterinary Medicine	Google Scholar
513	Subtype distribution of Blastocystis isolates from synanthropic and zoo animals and identification of a new subtype	Stensvold, C. R., Alfellani, M. A., Norskov-Lauritsen, S., Prip, K., Victory, E. L., Maddox, C., Nielsen, H. V., & Clark, C. G	2009	International Journal for Parasitology	Scopus
514	Determination of the effects of anti-parasitic drugs on the viability of Blastocystis strains via MTT assay.	Dogruman-Al, F., Adiyaman, G., Yantira, T. N., Hasgur, S., Bagriacik, E. U., & Sahin, I	2009	Kafkas Universitesi Veteriner Fakultesi Dergisi	Scopus
515	Blastocystis: unravelling potential risk factors and clinical significance of a common but neglected parasite.	Stensvold, C. R., Lewis, H. C., Hammerum, A. M., Porsbo, L. J., Nielsen, S. S., Olsen, K., Arendrup, M. C., Nielsen, H. V., & Mølbak, K	2009	Epidemiology and Infection	Web of Science
516	Subtype distribution of Blastocystis isolates from synanthropic and zoo animals and identification of a new subtype	Stensvold, C. R., Alfellani, M. A., Norskov-Lauritsen, S., Prip, K., Victory, E. L., Maddox, C., Nielsen, H. V., & Clark, C. G	2009	International Journal for Parasitology	Web of Science

S/N	Title	Authors	Year	Journal	Search Source
517	Genetic variability of Blastocystis sp. isolates obtained from cancer and HIV/AIDS patients	Tan TC, Ong SC, Suresh K	2009	Parasitology Research	Google Scholar
518	Pursuing the clinical significance of Blastocystis-diagnostic limitations	Stensvold, C. R., Nielsen, H. V., Mølbak, K., & Smith, H. V	2009	Trends in Parasitology	PubMed
519	Association of Blastocystis subtype 3 and 1 with patients from an Oregon community presenting with chronic gastrointestinal illness	Jones MS, Whipps CM, Ganac RD, Hudson NR, Boorom K	2009	Parasitology Research	PubMed
520	Phylogenetic analysis of Blastocystis isolates from animal and human hosts in the Philippines	Rivera WL	2008	Vet Parasitol	Web of Science
521	Drinking water: a possible source of Blastocystis spp. subtype 1 infection in schoolchildren of a rural community in central Thailand	Leelayoova S, Siripattanapipong S, Thathaisong U, Naaglor T, Taamasri P, Piyaraj P, Mungthin M	2008	Am J Trop Med Hyg	Google Scholar
522	Predominance of subtype 3 among Blastocystis isolates from a major hospital in Singapore.	Wong KHS, Ng GC, Lin RTP, Yoshikawa H, Taylor MB, Tan KSW	2008	Parasitology Research	Web of Science
523	Molecular epidemiology of Blastocystis infections in Turkey	Özyurt, M., Kurt, Ö., Mølbak, K., Nielsen, H. V., Haznedaroglu, T., & Stensvold, C. R	2008	Parasitology International	Google Scholar
524	Treatment-ladder and genetic characterisation of parasites in refractory giardiasis after an outbreak in Norway	Mørch, K., Hanevik, K., Robertson, L. J., Strand, E., & Langeland, N	2008	Journal of Infection	PubMed
525	Treatment-ladder and genetic characterisation of parasites in refractory giardiasis after an outbreak in Norway	Mørch, K., Hanevik, K., Robertson, L. J., Strand, E., & Langeland, N	2008	Journal of Infection	Web of Science
526	Oh my aching gut: irritable bowel syndrome, Blastocystis, and asymptomatic infection	Boorom, K. F., Smith, H. V., Nimri, L. F., Viscogliosi, E., Spanakos, G., Parkar, U., Li, L. H., Zhou, X. N., Ok, U. Z., Leelayoova, S., & Jones, M. S	2008	Parasites & Vectors	Google Scholar

S/N	Title	Authors	Year	Journal	Search Source
527	Detecting Blastocystis using parasitologic and DNA-based methods: a comparative study	Stensvold, C. R., Arendrup, M. C., Jespersgaard, C., Molbak, K., & Nielsen, H. V	2007	Diagnostic Microbiology and Infectious Disease	Scopus
528	Direct characterization of Blastocystis from faeces by PCR and evidence of zoonotic potential.	Parkar U, Traub RJ, Kumar S, Mungthin M, Vitali S, Leelayoova S, Morris K, Thompson R	2007	Parasitology	Google Scholar
529	Detecting Blastocystis using parasitologic and DNA-based methods: a comparative study	Stensvold, C. R., Arendrup, M. C., Jespersgaard, C., Molbak, K., & Nielsen, H. V	2007	Diagnostic Microbiology and Infectious Disease	PubMed
530	Terminology for Blastocystis subtypes-a consensus	Stensvold C, Suresh G, Tan K	2007	Trends Parasitol	Scopus
531	The epidemiology of Blastocystis hominis in the United States	Amin OM	2006	Research Journal of Parasitology	Web of Science
532	The epidemiology of Blastocystis hominis in the United States	Amin OM	2006	Research Journal of Parasitology	PubMed
533	A Placebo-Controlled Treatment Trial of Blastocystis hominis Infection with Metronidazole	Nigro L, Larocca L, Massarelli L, Patamia I, Minniti S, Palermo F, Cacopardo B	2006	Journal of Travel Medicine	Google Scholar
534	Trichomonas vaginalis and trichomoniasis in the Republic of Korea	Ryu J, Min D	2006	Korean Journal of Parasitology	Scopus
535	Blastocystis hominis and the evaluation of efficacy of metronidazole and trimethoprim/sulfamethoxazole	Moghaddam DD, Ghadirian E, Azami M	2005	Parasitology Research	Web of Science

S/N	Title	Authors	Year	Journal	Search Source
536	Treatment of Giardiasis.	Petri WA	2005	Current Treatment Options in Gastroenterology	Google Scholar
537	Blastocystis hominis and travelers	Sohail MR, Fischer PR	2005	Travel Medicine and Infectious Disease	Web of Science
538	Effect of nitazoxanide in persistent diarrhea and enteritis associated with Blastocystis hominis	Rossignol J, Kabil SM, Said M, Samir H, Younis AM	2005	Clinical Gastroenterology and Hepatology	Scopus
539	Irritable bowel syndrome: in search of an etiology: role of Blastocystis hominis.	Yakoob J, Jafri W, Jafri N, Khan R, Islam M, Beg MA, Zaman V	2004	American Journal of Tropical Medicine and Hygiene	Web of Science
540	Molecular and phylogenetic analysis of Blastocystis isolates from various hosts	Abe N	2004	Veterinary Parasitology	Scopus
541	In vitro susceptibility of Blastocystis hominis isolated from patients with irritable Bowel syndrome	Yakoob J, Jafri W, Jafri N, Islam M, Beg MA	2004	British Journal of Biomedical Science	PubMed
542	Histophagous scuticociliatids (Ciliophora) parasitizing turbot Scophthalmus maximus: morphology, in vitro culture and virulence.	Alvarez-Pellitero, P., Palenzuela, O., Padros, F., Sitja-Bobadilla, A., Riaza, A., Silva, R., & Aran, J	2004	Folia Parasitologica	Scopus
543	Histophagous scuticociliatids (Ciliophora) parasitizing turbot Scophthalmus maximus: morphology, in vitro culture and virulence.	Alvarez-Pellitero, P., Palenzuela, O., Padros, F., Sitja-Bobadilla, A., Riaza, A., Silva, R., & Aran, J	2004	Folia Parasitologica	PubMed

S/N	Title	Authors	Year	Journal	Search Source
544	Evidence of waterborne transmission of Blastocystis hominis	Leelayoova S, Rangsin R, Taamasri P, Naaglor T, Thathaisong U, Mungthin M	2004	American Journal of Tropical Medicine and Hygiene	Scopus
545	Evaluación de la nitazoxanida en dosis única y por tres días en parasitosis intestinal [Nitazoxanide vs albendazole against intestinal parasites in a single dose and for three days]	Belkind-Valdovinos U, Belkind-Gerson J, Sanchez-Francia D, Espinoza-Ruiz MM, Lazcano-Ponce E	2004	Salud Publica De Mexico	Scopus
546	Histophagous scuticociliatids (Ciliophora) parasitizing turbot Scophthalmus maximus: morphology, in vitro culture and virulence.	Alvarez-Pellitero, P., Palenzuela, O., Padros, F., Sitja-Bobadilla, A., Riaza, A., Silva, R., & Aran, J	2004	Folia Parasitologica	PubMed
547	Evaluación de la nitazoxanida en dosis única y por tres días en parasitosis intestinal [Nitazoxanide vs albendazole against intestinal parasites in a single dose and for three days]	Belkind-Valdovinos U, Belkind-Gerson J, Sanchez-Francia D, Espinoza-Ruiz MM, Lazcano-Ponce E	2004	Salud Publica De Mexico	Google Scholar
548	Blastocystosis: nitazoxanide as a new therapeutic option	Cimerman S, Ladeira MCT, Iuliano WA	2003	Revista Da Sociedade Brasileira De Medicina Tropical	Scopus
549	Efficacy of antigiardial drugs	Wright JM, Dunn LA, Upcroft P, Upcroft JA	2003	Expert Opinion on Drug Safety	Scopus

S/N	Title	Authors	Year	Journal	Search Source
550	Blastocystosis: nitazoxanide as a new therapeutic option	Cimerman S, Ladeira MCT, Iuliano WA	2003	Revista Da Sociedade Brasileira De Medicina Tropical	PubMed
551	Phylogenetic analysis of Blastocystis isolates from different hosts based on the comparison of small-subunit rRNA gene sequences	Noël, C., Peyronnet, C., Gerbod, D., Edgcomb, V. P., Delgado-Viscogliosi, P., Sogin, M. L., Capron, M., Viscogliosi, E., & Zenner, L	2003	Molecular and Biochemical Parasitology	Scopus
552	A survey of Blastocystis sp. in livestock, pets, and zoo animals in Japan	Abe N, Nagoshi M, Takami K, Sawano Y, Yoshikawa H	2002	Veterinary Parasitology	Scopus
553	Blastocystis hominis: neutral red supravital staining and its application to in vitro drug sensitivity testing.	Vdovenko AA, Williams JE	2000	Parasitology Research	Scopus
554	Strain differences in Blastocystis isolates as detected by a single set of polymerase chain reaction primers	Init I, Mak JW, Hakim SL, Yong HS	1999	Parasitology Research	PubMed
555	Prevalence and clinical relevance of Blastocystis hominis in diverse patient cohorts	Cirioni O, Giacometti A, Drenaggi D, Ancarani F, Scalise G	1999	European Journal of Epidemiology	Scopus
556	Irritable Bowel Syndrome in Patients with Blastocystis hominis Infection	Giacometti A, Cirioni O, A F, Fortuna M, Scalise G	1999	European Journal of Clinical Microbiology & Infectious Diseases	Scopus

S/N	Title	Authors	Year	Journal	Search Source
557	Significantly Increased IgG2 Subclass Antibody Levels to Blastocystis hominis in Patients with Irritable Bowel Syndrome	Hussain R, Jaferi W, Zuberi S, Baqai R, Abrar N, Ahmed A, Zaman V	1997	American Journal of Tropical Medicine and Hygiene	Web of Science
558	Blastocystis hominis revisited	Stenzel DJ, Boreham PFL	1996	Clinical Microbiology Reviews	Scopus
559	Blastocystis hominis: prevalence in asymptomatic versus symptomatic hosts	Udkow MP, Markell EK	1993	The Journal of Infectious Diseases	Web of Science
560	Cloning of a virulence factor of Entamoeba histolytica. Pathogenic strains possess a unique cysteine proteinase gene.	Reed S, J B, Pollack AS, Engel JC, Brown M, Hirata K, Que X, Eakin A, Hagblom P, Gillin FD	1993	Journal of Clinical Investigation	PubMed
561	Blastocystis hominis--past and future	Zierdt CH	1991	Clinical Microbiology Reviews	Scopus
562	Blastocystis hominis: Epidemiology and Natural History	Senay H, MacPherson DW	1990	The Journal of Infectious Diseases	Web of Science
563	Epidemiology and pathogenicity of Blastocystis hominis	Doyle PW, Helgason MM, Mathias RG, Proctor EM	1990	Journal of Clinical Microbiology	PubMed
564	Epidemiology and pathogenicity of Blastocystis hominis	Doyle PW, Helgason MM, Mathias RG, Proctor EM	1990	Journal of Clinical Microbiology	Web of Science

S/N	Title	Authors	Year	Journal	Search Source
565	Epidemiology and clinical features associated with Blastocystis hominis infection	Kain KC, Noble MA, Freeman HJ, Barteluk RL	1987	Diagnostic Microbiology and Infectious Disease	PubMed
